# Dibutyl Itaconate and Lauryl Methacrylate Copolymers by Emulsion Polymerization for Development of Sustainable Pressure-Sensitive Adhesives

**DOI:** 10.3390/polym14030632

**Published:** 2022-02-07

**Authors:** Carlos Rafael Casas-Soto, Alain Salvador Conejo-Dávila, Velia Osuna, David Chávez-Flores, José Carlos Espinoza-Hicks, Sergio Gabriel Flores-Gallardo, Alejandro Vega-Rios

**Affiliations:** 1 Departament of Engineering and Materials Chemistry, Centro de Investigación en Materiales Avanzados, SC, Miguel de Cervantes No. 120, Chihuahua C.P. 31136, Mexico; carlos.casas@cimav.edu.mx (C.R.C.-S.); alain.conejo@cimav.edu.mx (A.S.C.-D.); sergio.flores@cimav.edu.mx (S.G.F.-G.); 2 Consejo Nacional de Ciencia y Tecnología (CONACyT)—Centro de Investigación en Materiales Avanzados, SC (CIMAV), Miguel de Cervantes No. 120, Chihuahua C.P. 31136, Mexico; velia.osuna@cimav.edu.mx; 3 Facultad de Ciencias en Química, Universidad Autonóma de Chihuahua, Chihuahua C.P. 31125, Mexico; dchavezf@uach.mx (D.C.-F.); jhicks@uach.mx (J.C.E.-H.)

**Keywords:** itaconic acid, di-*n*-butyl itaconate, lauryl methacrylate, pressure-sensitive adhesives, emulsion polymerization, renewable monomers, sustainable polymers

## Abstract

Renewable polymers possess the potential to replace monomers from petrochemical sources. The design and development of polymeric materials from sustainable materials are a technological challenge. The main objectives of this study were to study the microstructure of copolymers based on itaconic acid (IA), di-*n*-butyl itaconate (DBI), and lauryl methacrylate (LMA); and to explore and to evaluate these copolymers as pressure-sensitive adhesives (PSA). The copolymer synthesis was carried out through batch emulsion radical polymerization, an environmentally friendly process. IA was used in a small fixed amount as a functional comonomer, and LMA was selected due to low glass transition temperature (*T_g_*). The structure of synthesized copolymers was studied by FTIR, ^1^H-NMR, Soxhlet extraction, and molecular weight analyses by GPC. Furthermore, the viscoelastic and thermal properties of copolymer films were characterized by DMA, DSC, and TGA. The single *T_g_* displayed by the poly(DBI-LMA-IA) terpolymers indicates that statistical random composition copolymers were obtained. Moreover, FTIR and NMR spectra confirm the chemical structure and composition. It was found that a cross-linked microstructure and higher molecular weight are observed with an increase of LMA in the feed led. The *T_g_* and modulus (G′) of the copolymers film can be tuned with the ratio of DBI:LMA providing a platform for a wide range of applications as a biobased alternative to produce waterborne PSA.

## 1. Introduction

The development of polymeric materials from renewable resources has become essential in recent years due to the depletion of natural and fossil resources. Additionally, growing environmental awareness and more stringent regulations on the volatile organic compounds content in coatings and adhesives are the major driving forces for developing environmentally friendly processes or materials [1,2,3]. Currently, the majority of commercial pressure-sensitive adhesives (PSA) materials are acrylic-based, derived from fossil resources, due to their high performance [4]. The term PSA refers to a viscoelastic material that adheres to a solid surface “permanently” at room temperature, under slight pressure, and short contact time without any phase transition or chemical reaction [5]. The widespread use of PSA in everyday life is immense, for example, labels, tapes, sticky notes, bandages, patches, and protective films [6]. Nevertheless, the commercial PSAs interfere with the recycling industry and its product quality, e.g., paper recycling. Consequently, there are challenges in replacing these materials for biobased polymers [6].

On the other hand, emulsion polymerization is a typical process for obtaining polymeric particles dispersed in a liquid medium, usually water [7]. The use of water as a dispersed medium has been considered an environmentally friendly process and is ideal for polymerizing biobased monomers [7]. For example, the synthesis of protective coatings based on branched vinyl esters [8], self-crosslinkable prepolymers of lauryl methacrylate with trimethylolpropane trimethacrylate [9], and the synthesis of terpene-based methacrylate copolymer [10].

Moreover, several studies on sustainable water-based PSA produced by emulsion polymerization have been published for these reasons. For instance, Droesbake et al. [11] obtained fully biobased waterborne PSA from terpenoid-based (meth)acrylates that showed similar adhesive properties compared to a conventional PSA and were classified within general-purpose and high shear PSAs according to the viscoelastic windows (VW). Badía and coworkers [12] developed PSAs with high biobased content (up to 72%) based on isobornyl methacrylate (derived from terpenes of pine oil) and 2-octyl acrylate (derived from vegetable castor oil). They found that directly replacing the oil-based monomers with renewable monomers did not provide yielded identical adhesive performance due to differences in the polymer microstructure requiring formulation optimization. Molina-Gutierrez et al. reported the polymerization of methacrylated eugenol via emulsion with adhesive properties [3,13]. Therefore, there are different sources for synthesizing monomers and their subsequent polymerization, such as lignin [14], carbohydrate derivatives, plant oils, and terpenes [10].

IA is an unsaturated aliphatic dicarboxylic acid obtained by the fermentation of carbohydrates using certain filamentous fungi [15,16] that can be polymerized through free radical polymerization or via polycondensation [17,18]. IA has been extensively used as a functional monomer in emulsion polymerization to improve colloidal stability and substrate adhesion [19]. In the biopolymers field, IA and its derivates hold promise for providing sustainable materials owing to its versatility, renewable nature, commercial availability, and high potential to replace petroleum-derived monomers specifically, acrylic acid, methacrylic acid, and maleic anhydride [20,21]. Likewise, DBI is a biobased monomer derived from the esterification of IA and 1-butanol [21]. In addition, DBI is mainly utilized as a comonomer in the synthesis of biobased elastomers [19,22]. Lastly, LMA is a fatty acid-based monomer that provides low glass transition temperatures (*T_g_*) into a polymer film. Therefore, it is an excellent substitute for replacing low *T_g_* monomers, for example, butyl acrylate and 2-ethylhexyl acrylate, in different industrial applications [20]. In addition, to flexibility, hydrophobicity, and improved adhesion to low-energy surfaces [23].

The importance and originality of this study are that it explores, for the first time, the synthesis of copolymers via emulsion polymerization from biobased monomers—itaconic acid (IA), di-*n-*butyl itaconate (DBI), and lauryl methacrylate (LMA). Furthermore, the synthesized copolymers were designed with the final application of the PSA type. Typically, an acrylate PSA contains a monomer with acid functional groups (3–10 wt %) and a soft polymer (90–97 wt %) [4]. Hence, in this study, the copolymers contain IA at 1 wt %, and the soft polymer is made up of a DBI:LMA ratio with a total sum of 99 wt %.

In the present study, the main objectives of this study were to: (1) study the microstructure of copolymers based on itaconic acid (IA), di-*n*-butyl itaconate (DBI), and lauryl methacrylate (LMA); (2) explore these copolymers as pressure-sensitive adhesives (PSA); (3) to examine the effect of the monomer ratio (DBI: LMA) of synthesized copolymers on viscoelastic and *T_g_* properties.

## 2. Materials and Methods

### 2.1. Materials

The substances such as di-*n*-butyl itaconate (DBI), lauryl methacrylate (LMA), itaconic acid (IA), sodium dodecyl sulfate (SDS) surfactant, potassium persulfate (KPS) initiator, and hydroquinone (HQ) inhibitor were acquired from Sigma-Aldrich (Saint Louis, MO, USA). In addition, ethanol, petroleum ether, sodium hydroxide (NaOH), and ammonia (28 wt % in H_2_O) were obtained from Fermont (Monterrey, Nuevo Leon, Mexico). The degree of purity of the above substances was of reagent grade. Deionized water (DI-H_2_O) was used throughout the investigation. Tetrahydrofuran (THF, HPLC grade from JT Baker (Phillipsburg, NJ, USA)) solvent was used in the polymer characterization. Argon gas (Praxair, Danbury, CT, USA) was used to purge the reactor. Cellulose extraction thimbles (Whatman^™^, Marlborough, MA, USA) were used in gel content measurements. DBI and LMA were purified prior to polymerization, passing them through an inhibitor removal column of basic alumina or Brockmann I (Sigma-Aldrich, Saint Louis, MO, USA). SDS was purified by Soxhlet extraction with petroleum ether for 24 h, followed by recrystallization with absolute ethanol. All other chemicals were used as supplied.

### 2.2. Polymer Latex Preparation

Latex dispersions were synthesized via the batch emulsion polymerization process, specifically, in a three-neck glass reactor (100 mL) equipped with a mechanical stirrer, thermometer, and argon supply inlet. The formulations are shown in Table 1.

The latexes, for instance PSA-2000, were prepared under the following method. Firstly, SDS surfactant (0.95 g, 24 parts per hundred parts of monomer (phm)) was dissolved in DI-H_2_O in a beaker. Meanwhile, in another beaker, DBI (16.9 g), LMA (5.7 g), and IA (0.23 g) were stirred magnetically for 30 min. The monomers mix was then poured into the SDS solution and stirred intensely for 30 min. The emulsification process was complemented by ultrasound sonification because LMA is extremely hydrophobic. Afterward, the emulsion was transferred to the reactor, which was placed in a thermostated oil bath and mechanically stirred at 300 rpm under the argon atmosphere. The aqueous initiator solution (KPS (0.23 g, 1.0 phm) dissolved in 5 g of deoxygenated DI-H_2_O) was injected quickly into the reactor. The copolymerization time was 6 h at a temperature of 75 °C. Finally, the latex was filtered with a nylon fabric (80 mesh) to remove any coagulum formed during the copolymerization, and the pH was adjusted to ~8 to prevent hydrolysis. The final solid content was between 20 and 25 wt %.

### 2.3. Caracterization of Copolymer Latex

The degree of monomer conversion was determined gravimetrically. First, 1 mL of latex sample was withdrawn from the reactor and transferred to an aluminum dish. Subsequently, 1 mL of a hydroquinone solution (0.1 wt %) was added and weighed. Then, the samples were dried at 65 °C for 24 h in a convection oven and, finally, utilizing a vacuum oven at 40 °C until constant weight.

Particle size was measured by dynamic light scattering (DLS) with Malvern Zetasizer Nano S (Malvern Panalytical, Almelo, The Netherlands) equipment with an angle of 173°. For the analysis, the latex was diluted with DI-H_2_O at 0.1 wt %. The reported diameter is the intensity-weighted average particle size of three measurements.

### 2.4. Preparation and Characterization of Copolymer Films

#### 2.4.1. Preparation of Copolymer Films

Copolymer films were prepared by casting latex on silicon molds controlling the thickness to 1 ± 0.1 mm. First, the latexes were poured into their respective mold drying for 7 days at ambient temperature (20 °C) inside a closed chamber. Then, the films and their molds were transferred to a convection oven for subsequent drying for 2 days at 45 °C. Finally, the films were dried to a constant weight utilizing a vacuum oven at 35 °C. It is important to note that the vacuum was slowly increased to prevent the formation of bubbles on the surface of the film due to traces of water.

#### 2.4.2. Copolymer Film Characterization

NMR experiments were performed on a Bruker Advanced III 400 MHz (Billerica, MA, USA) spectrometer. The copolymers films were studied utilizing deuterated chloroform (CDCl_3_) at a temperature of 25 °C. ^1^H NMR (using PROTON pulse sequence, NS = 16, and D1 = 10) estimated the copolymer composition, and the elucidation of copolymer structure was supported by ^13^C NMR (NS = 1024), homonuclear correlation spectroscopy, usually called COSY, and heteronuclear single-quantum correlation (HSQC) and distortionless enhancement by polymerization transfer (DEPT-135) analysis.

The Fourier transform infrared spectroscopy (FT-IR) spectra of copolymer films were recorded in a Shitmatzu IR Affinity-1S (Kyoto, Japan) spectrophotometer, equipped with an ATR diamond accessory. In addition, the samples were analyzed from 4000 cm^−1^ to 500 cm^−1^ with a resolution of 4 cm^−1^.

The gel content of the synthesized copolymers was determined by Soxhlet extraction using THF under reflux for 24 h. Afterward, the samples were dried until a constant weight was achieved. The gel content was calculated as the ratio between the non-extracted polymer (W_g_) and the initial polymer sample (W_p_). The reported value is the average of three measurements.

The polymer molecular weight (M_w_) and molecular weight distribution (MWD) of the remaining solution from the gel content determination were measured by size exclusion chromatography employing a Chromatograph PL-GPC 220 Agilent (Santa Clara, CA, USA). The equipment was calibrated with polystyrene standards.

Differential scanning calorimetry (DSC) analysis of copolymer films was performed utilizing a TA instruments DSC Q200 (TA Instrument, New Castle, DE, USA) equipped with a TA-TCS90 cooling system. The copolymer films were studied at a heating rate of 10 °C/min with an argon atmosphere (100 mL/min). The cycle consists of four steps: (i) heating from 25 to 170 °C (10 °C/min); (ii) isothermal for 5 min at 170 °C; (iii) cooling from 170 to –90 °C (10 °C/min); and (iv) heating from –90 to 100 °C (10 °C/min). Glass transition temperatures (*T_g_*) were calculated utilizing the second scan.

Thermogravimetric analysis (TGA) of copolymer films was carried out using TA instruments TGA/SDT Q600 equipment (TA, Instrument, New Castle, DE, USA). Thermograms were carried out from 25 °C to 800 °C in an argon atmosphere at a 50 mL/min flow rate. The heating ramp was 10 °C/min.

The linear viscoelastic properties of the copolymer films were characterized by Anton Paar Physica MCR 501 rheometer (Graz, Austria) with 25 mm parallel plate geometry and at a temperature of 25 °C. The thickness of the samples was 1.0 mm ± 0.1 mm. First, a strain sweep at a constant frequency of 1 Hz was done to determine the viscoelastic linear region. Then, a frequency sweep was carried out at room temperature in a range from 0.01 to 100 Hz at 0.5% strain. All samples were performed in triplicate.

### 2.5. Adhesive Properties of Copolymers

A Universal Instron tester model 4469 with a 100 N load cell (cat no. 2525-807) was used to evaluate loop tack, peel strength, and shear strength. The copolymer films were prepared by coating on PET sheet (50 μm, 2 mils) with a bar-type applicator (BYK model 5306) with a gap of 152 μm (6 mils). The thickness of copolymer films were around 30 μm; drying at 60 °C in a convection oven for an hour and finally covered with silicone paper. Previously to tests, the copolymer films were conditioned for 24 h at a temperature of 25 °C and relative humidity of 50%.

Peel strength was determined at a 180° peel angle according to ASTM D3330 test method A. In addition, the copolymer films were cut with rectangular shapes with the following dimensions of 25.4 × 270 mm. The sample was then laminated on stainless steel or polypropylene with a roller (2 kg) with a dwell time of 1 min. The test fixture was placed in the tensile tester’s lower grip, and the strip’s free end was clamped to the upper jaw, which pulled at a constant crosshead speed of 300 mm/min. The average force required to peel away the tape was recorded; three replicates of each sample were tested, and the average force was reported along with the failure mode.

A sample (25.4 × 250 mm) was cut and bent into a loop with the adhesive surface facing outward for the tack test. The ends of the sample were fastened with 25.4 mm masking tape and placed in the upper grip of the tensile tester. Afterward, the upper grip was manually moved downward until the sample loop covered the 25.4 mm area of the stainless-steel fixture completely. It subsequently moved immediately upwards with a cross speed of 300 mm/min. The maximum force and the mode of failure were recorded. The method was performed according to ASTM D6195-03 test method A.

A sample was prepared for the shear strength (ASTM D3986-19) testing from casting an adhesive film of 200 µm thick on 25.4 mm wide aluminum. Prior to the test, the sample was bonded to another aluminum forming a single lap joint, with a dwell time of 20 min. The cross speed was set at 1 mm/min. The shear area was 25.4 × 25. 4 mm. The average shear strength of three samples was taken from the ultimate tensile strength of the stress–strain diagram.

## 3. Results and Discussion

The emulsion polymerization products involve the copolymerization of two or more monomers incorporated into polymer chains. Under the provided synthesis conditions, the polymerization mechanism determines the characteristics of the polymer—e.g., composition and microstructure—significantly influencing the end-use properties of the polymers [24,25]. The classic emulsion polymerization has three stages or intervals, including nucleation period (I), particle growth at a constant rate of polymerization (II), and particle growth absence of monomer droplet decreases the rate of polymerization (III). The particle nucleation (Figure S1), interval I, is classified into three mechanisms: micellar nucleation, homogeneous nucleation, and droplet nucleation. Especially for these systems, ab initio emulsion polymerization contains a surfactant concentration above the critical micellar concentration. Thus, the micellar nucleation occurs primarily under this condition. In addition, the copolymerization is initiated by persulfates, where the radicals are generated in the aqueous phase. These actives species react with monomer molecules in the aqueous phase creating oligomeric radicals, which the monomer-swollen micelles can capture [26].

On the other hand, a vital aspect to consider according to the final application is the selection of monomers, particularly pressure-sensitive adhesives (PSA). The selected monomers were based on physicochemical characteristics—e.g., di-*n*-butyl itaconate (DBI) as the medium glass transition temperature (*T_g_*) monomer, lauryl methacrylate (LMA) as a low *T_g_* monomer, and itaconic acid (IA) as a functional monomer [27].

### 3.1. NMR Spectroscopy

**Figure 1:**
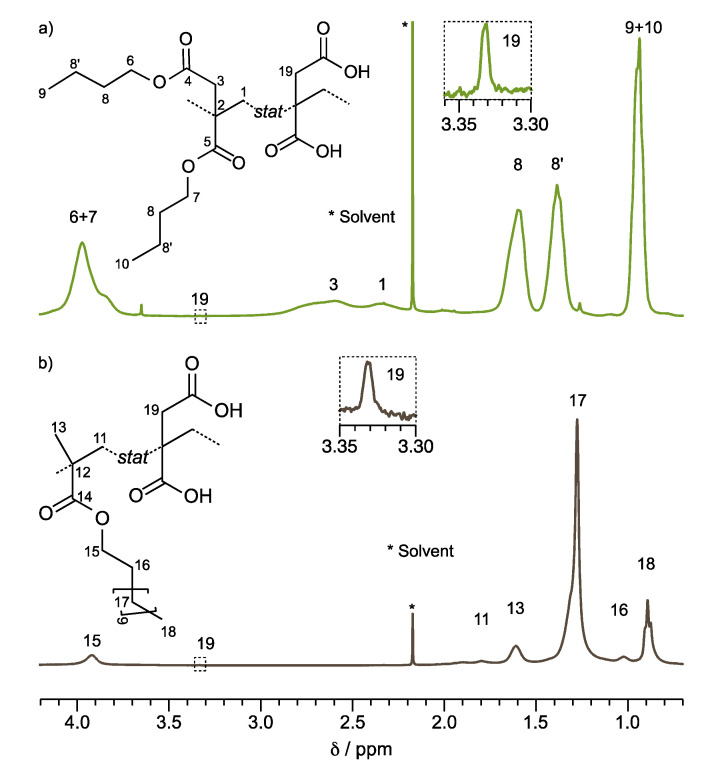
^1^H-NMR spectra of films copolymers in CDCl_3_. (**a**) PSA-1000 (Poly(DBI_99_-*stat-*IA_1_)); (**b**) PSA-5000 (Poly(LMA_99_-*stat-*IA_1_)).

The structure elucidation of the PSA-1000 (Poly(DBI_99_-*stat-*IA_1_)] and PSA-5000 [Poly(LMA_99_-*stat-*IA_1_)) copolymers was determined by ^1^H NMR. Figure 1a,b illustrate the ^1^H NMR spectra of PSA-1000 and PSA-5000, respectively. These copolymers contain DBI or LMA at 99 wt %, so the assignment of the signals corresponding to their chemical shift is facilitated. Figure 1a, the signal corresponding the protons of α-methylene to the carbonyl of the ester 3 (2H) and the main chain 1 (2H) are located between δ = 1.8 and 3.2 ppm. In addition, the butyl substituents have a similar chemical environment, so their signals overlap, and it is impossible to differentiate one from the other. Nevertheless, it is possible to observe that the proton signals 6 and 7 correspond to the α-methylene to the oxygen of the ester. These protons are located at a low field, around δ = 4 ppm (4H), because of the inductive effect provided by the neighboring oxygen. On the other hand, the terminal methyl’s (9 and 10) signal of the butyl substituent is observed at δ = 0.95 ppm (6H). The methylene of the butyl chain named 8 and 8′ are observed at δ = 1.6 and 1.4 ppm, respectively. Figure S2 displays the PSA-1000 spectrum ^1^H NMR integrated and assigned with identical nomenclature. According to the present results, Satoh et al. [28] reported an identical assignment of proton signals for a DBI copolymer; however, other similar works report the methylene protons 1 of the main chain at δ = 1.4 [29] and 1.6 [30].

The spectrum of PSA-5000 (Poly (LMA_99_-*stat*-IA_01_)), Figure 1b, presents a broad signal (2.1 to 1.7 ppm (2H)) assigned to the methylene of the main chain 11. The singlet belonging to methyl 13 is also observed at 1.6 ppm (3H). The signals that confirm the dodecyl substituent are located at 0.9 ppm, triplet (3H), corresponding to the methyl protons 18; the singlet at 1.3 ppm (18H) corresponding to the methylene 17; and the signals of the methylenes 15 and 16 are observed at 3.9 (2H) and 1.0 (2H) ppm, respectively [31]. Figure S3 displays the PSA-5000 ^1^H NMR spectrum integrated and assigned with identical nomenclature.

The ternary copolymers PSA-2000 (Poly(DBI_74_-*stat*-LMA_25_-*stat*-IA_01_)), PSA-3000 (Poly(DBI_49_-*stat*-LMA_50_-*stat*-IA_01_)), and PSA-4000 (Poly(DBI_24_-*stat*-LMA_75_-*stat*-IA_01_)) are shown in Figure 1b–d, respectively. The chemical shifts assigned in the ternary copolymers were based primarily on PSA-1000 and PSA-5000, e.g., PSA-3000 (Figure S4). The structural characterization was verified by different NMR techniques, such as two-dimensional homonuclear correlation spectroscopy ^1^H-^1^H COSY, heteronuclear single-quantum correlation ^1^H-^13^C HSQC, and distortionless enhancement by polymerization transfer (DEPT, only for PSA-3000). The ^1^H-^1^H COSY spectra of copolymers (Figures S5–S7) show the proposed signals correctly. For example, the comonomer LMA, specifically its aliphatic chain represented with peaks 18 (–CH_3_), 17 (–CH_2_–), 16 (–CH_2_–), and 15 (–O–CH_2_), present a correlation between them. Likewise, the butyl chain (DBI) has crossing points, including the methyl’s protons 9 and 10 with 8′ (–CH_2_–); the methylene’s 8′ correlated with 8; and the protons 8 couples protons around δ = 4 ppm—i.e., the protons signals 6 and 7 correspond to the α-methylene to the oxygen of the ester. In addition, the methylene’s protons of the main chain (DBI comonomer) are located close to 2.4 ppm without any correlation. Finally, the protons of α-methylene to the carbonyl of the ester 3 (2H) are also observed in the proposed region.

Additionally, ^1^H-^13^C HSQC (Figures S8–S10) study requires first assigning ^13^C NMR, which are shown below. PSA-3000 ^13^C NMR (CDCl_3_ 400 MH_z_), Figure S11, δ-207 ppm (4, 5, 14), 65.0 (15), 63.7 (6 + 7), 49 (1, 3, 11), 31.9 (17), 30.9 (12), 30.6 (8), 30.1 (8), 29.7 (17), 29.3 (17), 28.1 (17), 26.0 (13), 22.7 (17), 19.27 (8′), 14.1 (18), and 13.7 (9, 10). A DEPT-135 study was needed to differentiate CH, CH_2_, and CH_3_, Figure S12. The correlation of ^1^H and ^13^C NMR allows verifying that the carbon’s proposed displacements are correct. Therefore, the chemical structure of copolymers studied under NMR is confirmed.

**Figure 2:**
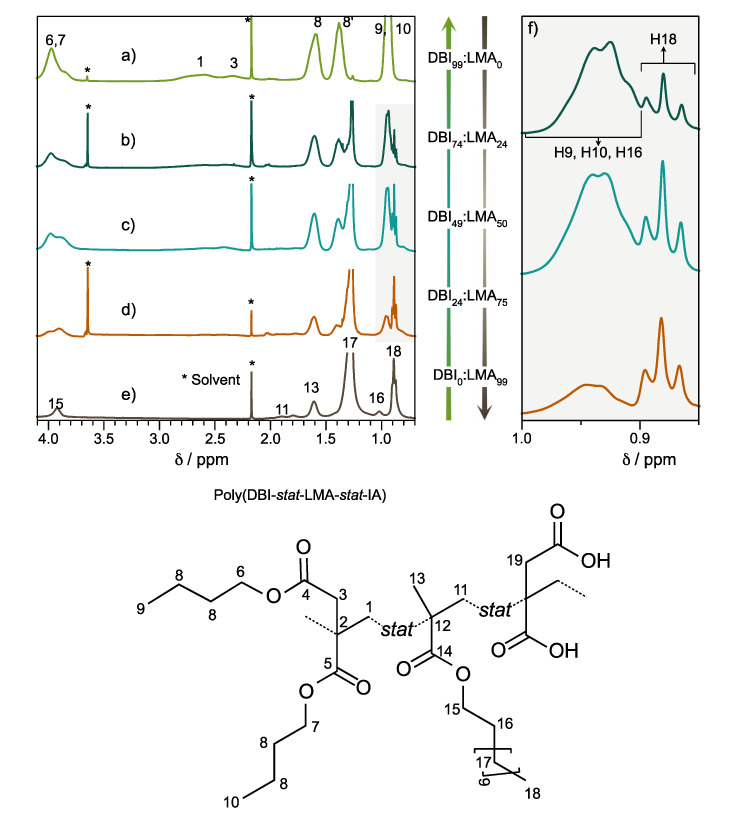
^1^H-NMR spectra of films copolymers in CDCl_3_. (**a**) PSA-1000; (**b**) PSA-2000 (Poly(DBI_74_-*stat*-LMA_25_-*stat*-IA_01_)); (**c**) PSA-3000 (Poly(DBI_49_-*stat*-LMA_50_-*stat*-IA_01_)); (**d**) PSA-4000 (Poly(DBI_24_-*stat*-LMA_75_-*stat*-IA_01_)); (**e**) PSA-5000; (**f**) Expansion of the region δ = 0.8–1.05 ppm of the copolymers PSA-2000, PSA-3000, and PSA-4000.

The intensity ratio and chemical shift of the peaks at δ = 0.89 and δ = 0.95 ppm did not change at different relaxation times (D1, Figure S13), indicating differences in relation to integration owing to different ratios DBI:LMA monomers, Figure 2. The signals, particularly at 0.89 and 0.95 ppm, corresponding to protons H_9_, H_10_, H_16_, and H_18_, can determine the ratio of DBI and LMA present in each compound, Table 2. About IA monomer was determined using the integration of α-methylene to the carbonyl of the ester localized at 3.3 ppm. Table S1 shows the areas and protons for calculating copolymer compositions.

### 3.2. FT-IR Spectroscopy

**Figure 3:**
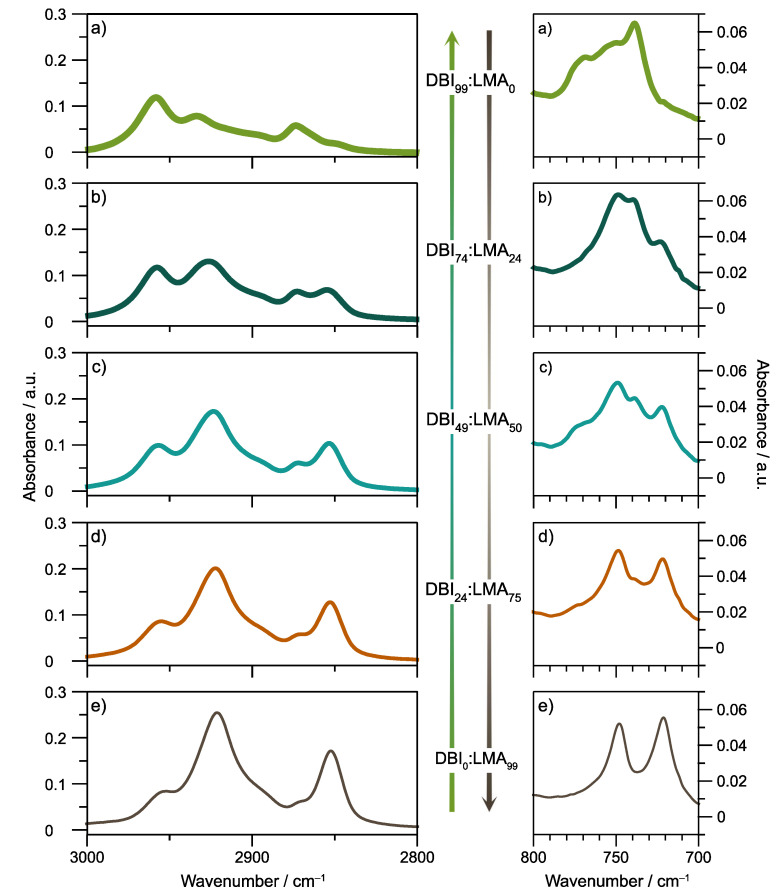
FTIR spectra of copolymers synthesized. (**a**) PSA-1000; (**b**) PSA-2000; (**c**) PSA-3000; (**d**) PSA-4000; (**e**) PSA-5000.

The synthesized copolymers were characterized by FT-IR spectroscopy to determine their chemical composition, analyzing the characteristic group frequencies. Figure 3 focuses on two regions of interest of the FT-IR absorption spectra of the copolymer films (the full spectra between 600 and 4000 cm^−1^ corresponding to the mid-IR region shown in the Supplementary Material). The first is the 2800–3000 cm^−1^ region; the peaks at 2923 and 2853 cm^−1^ are assigned to carbon-hydrogen (C–H) asymmetric and symmetric stretches, respectively, from the methylene group (–CH_2_–) of the alkyl substituents of both DBI and LMA. Similar to the NMR characterization, those peaks are the most representative to identify the presence of LMA in the different copolymers because their intensity is directly related to LMA content in the copolymer. Figures S14 and S15, the peak area at 2923 and 2853 cm^−1^, was correlated to the content of the LMA obtained by NMR, respectively. The bands at 2957 and 2874 cm^−1^ are assigned to the methyl group’s symmetric and asymmetric stretching vibrations of C–H bonds [32].

Secondly, at the low-frequency region due to the lauryl group, the methylene in phase rocking vibration 721 cm^−1^ increases depending on the content of the LMA repeat unit [33]. Lastly, Figure S16 of the supplementary material shows the entire spectra of all samples.

### 3.3. Molecular Weight, Gel Content, Conversion, and Particle Size

**Figure 4:**
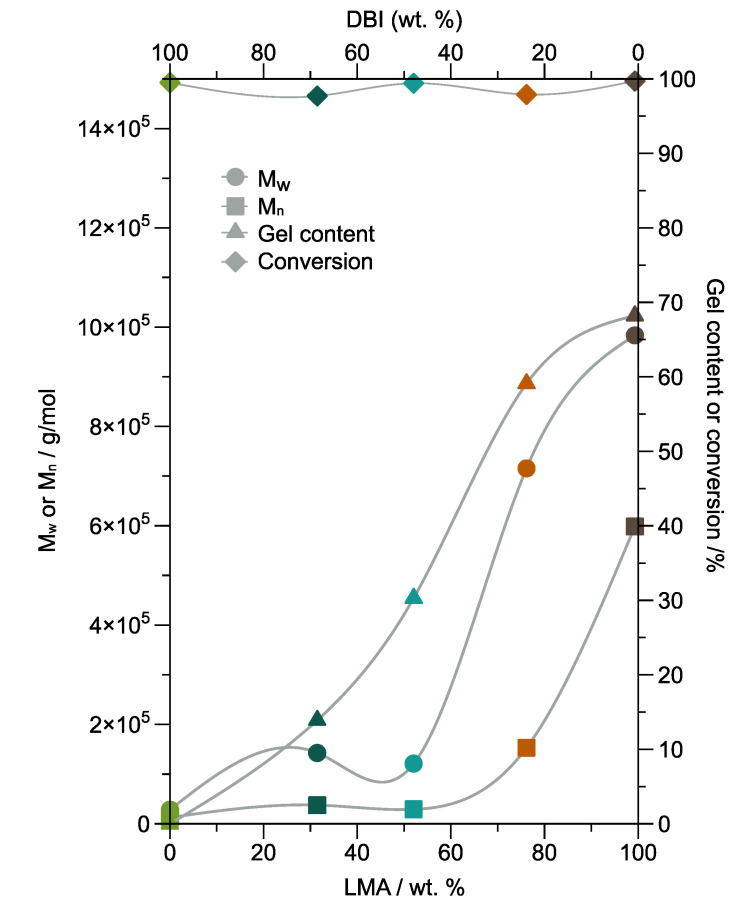
Graph of M_w_ (weight average molecular weight), M_n_ (number average molecular weight), gel content, and conversion values of the PSA-1000, PSA-2000, PSA-3000, PSA-4000, and PSA-5000 samples.

One of the parameters that affect the intrinsic properties of adhesives is the polymer microstructure [34]. For this reason, the molecular weight, gel content, and conversion of the five synthesized copolymers are studied. Figure 4 shows the weight average molecular weight (M_w_), number average molecular weight (M_n_), gel content, and conversion as DBI or LMA content function. The gel content measures the percentage of insoluble polymer fraction in THF, indicating the formation of the cross-linked polymer with high molecular weight [35]. The cross-linking degree also demonstrates the cohesive strength of the material [35]. In Figure 4, the results demonstrate that the major contributor to gel formation was LMA with 68% gel for PSA-5000. In contrast, PSA 1000 did not present the gel formation. Furthermore, the gel creation is from 10 min for PSA-4000, according to the kinetic study illustrated in Figure S17.

Moreover, methacrylic monomers mainly terminate by disproportionation, resulting in a polymer containing chain-end double bonds [36]. The emulsion polymerization process can occur chain branching and gel formation, without a cross-linking agent, due to the propagation of terminal double bond or via intermolecular chain transfer to polymer [37,38]. Furthermore, it has been reported that the polymerization of LMA by the mechanism free radical polymerization forms cross-linked networks at high conversion and temperatures up to 90 °C as a consequence of chain transfer to the polymer through the attack of the alkyl side-group [39,40]. In this research, the conversion from monomer to copolymer is above 97% for all samples. Consequently, the formation of a branched structure and cross-linked network is possible for PSA-2000, PSA-3000, PSA-4000, and PSA-5000 due to chain transfer to polymer, specifically the LMA.

**Figure 5:**
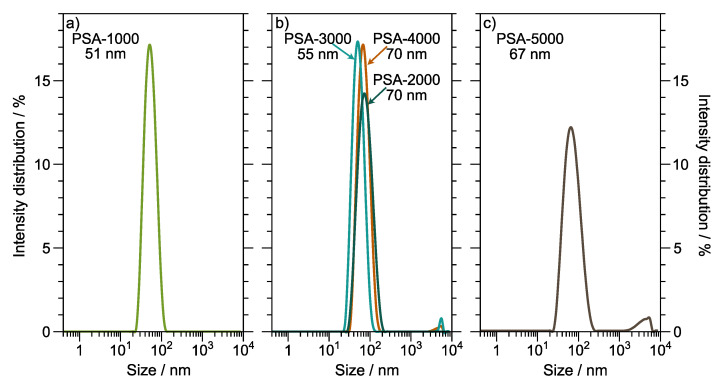
Particle size of copolymer latexes. (**a**) PSA-1000; (**b**) PSA-2000, PSA-3000, and PSA-4000; (**c**) PSA-5000.

Figure 5 shows the particle size of the synthesized copolymers. Latex has a particle size between 51 nm to 70 nm, suggesting control of emulsion polymerization conditions. Batch and semi-batch emulsion polymerization processes produce latex with particle size distribution typical 100 nm. However, the particle size distribution is influenced by several factors, such as the technique employed to disperse the monomer in the aqueous phase [41] and the type and concentration of the initiator [42].

### 3.4. Thermal Properties

#### 3.4.1. Glass Transition Temperature

The effect of the copolymer ratio (DMA:LMA) on *T*_g_ was investigated. This physicochemical property is one significant factor affecting the adhesive properties [43]. Figure S18 illustrates the thermograms of PSA-1000, PSA-2000, PSA-3000, PSA-4000, and PSA-5000. The PSA-1000 sample has a *T_g_* of 19 °C, and for PSA-5000, *T_g_* was not possible to observe; nevertheless, it displayed an endothermic melting peak around –28 °C associated with crystal domains arising from the crystallization of the long alkyl side chains of the LMA. These results coincide with the data reported for poly(LMA) [44,45]. The increase in the proportion of LMA in copolymers (PSA-2000 < PSA-3000 < PSA-4000) produces a decrease in the *T_g_* value, specifically at –10 °C, –23.5 °C, and –53 °C, respectively. These values are close to those calculated with the Fox equation [46] from the copolymer composition supply by NMR, Table 3.

Generally, copolymers produced by batch emulsion polymerization tend to display a composition drift in the polymer chains. In addition, a heterogeneous structure is generated owing to differences in the copolymerization reactivity ratios and monomer solubility in the aqueous phase, which leads to phase separation and exhibits more than one *T_g_* value [47]. The single *T_g_* value displayed by the Poly(DBI_X_-*stat*-LMA_Y_-*stat*-IA_1_) copolymer series suggests that the copolymerization proceeded statistically random, producing homogeneous composition polymers [48]. Another indication of random copolymerization is that the terpolymers do not show a melting peak. A possible explanation for this might be that incorporating DBI statically distributed changes the conformation in the polymer backbone, disrupting the order and preventing the crystallization of side chains. This phenomenon has also been reported for other types of copolymers, for example, in EVA copolymers, when vinyl acetate reaches a content of more than 45%, the structural polyethylene crystal phase disappears, or in the case of PLA, when D-lactide monomer is above 10 mol %, disturbs chain order and becomes fully amorphous [49,50].

#### 3.4.2. Thermal Degradation

**Figure 6:**
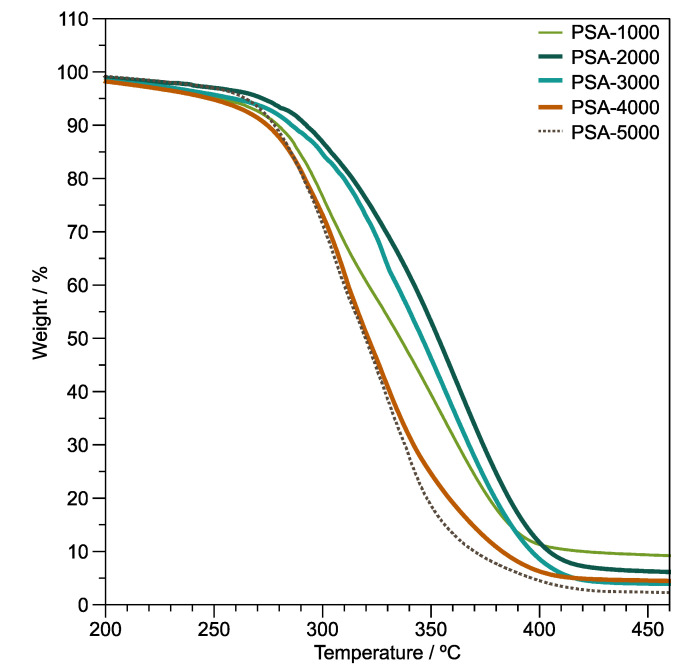
Thermogravimetric curves of PSA-1000, PSA-2000, PSA-3000, PSA-4000, and PSA-5000 films. Heating ramp = 10 °C/min, in an inert atmosphere.

Thermogravimetric analysis (TGA) under a non-oxidative atmosphere and non-isothermal conditions was used to assess the thermal stability of polymer films. Figure 6 displays thermogravimetric curves of the copolymer films. The thermograms of the PSA-1000, PSA-2000, PSA-3000, PSA-4000, and PSA-5000 samples present 2 stages of degradation. The first stage, 160–240 °C, could be attributed to the loss of volatile low molecular weight compounds and oligomers. In addition, the degradation temperature at 5% (*T_5%_*) of weight loss of PSA-1000, PSA-2000, PSA-3000, PSA-4000, and PSA-5000 samples are 250, 262, 259, 248, and 264 °C, respectively. In the second stage (T > 240 °C), depolymerization occurs during non-oxidative thermal degradation of the poly(di-*n*-alkyl itaconates) according to Popović et al. [51]. Furthermore, the thermal degradation of poly(*n*-alkyl methacrylates) is similar to the thermal degradation of the poly(di-*n*-alkyl itaconates) [51]. In fact, their chemical structures’ similarity allowed the establishment of the mechanisms of depolymerization of the poly(di-*n*-alkyl itaconates) [50,51]. On the other hand, a comparison of the degradation temperature at 50% (*T_50%_*) weight loss (Figure 6) of the ternary copolymers presents an improvement in thermal stability depending on the DBI content. This thermal stability behavior is typical of the poly(di-*n*-alkyl itaconates), which decreases with the increase of the alkyl group [52]. Likewise, Ghosh et al. [53] found that the thermal stability of the poly(lauryl methacrylate) diminished, compared with their poly(lauryl methacrylate)-*co*-poly(vinyl acetate) copolymers. The *T_50%_* values of PSA-1000, PSA-2000, PSA-3000, PSA-4000, and PSA-5000 samples are 336, 353, 346, 321, and 319 °C, respectively.

### 3.5. Viscoelastic Properties

#### 3.5.1. Oscillatory Frequency Sweep

**Figure 7:**
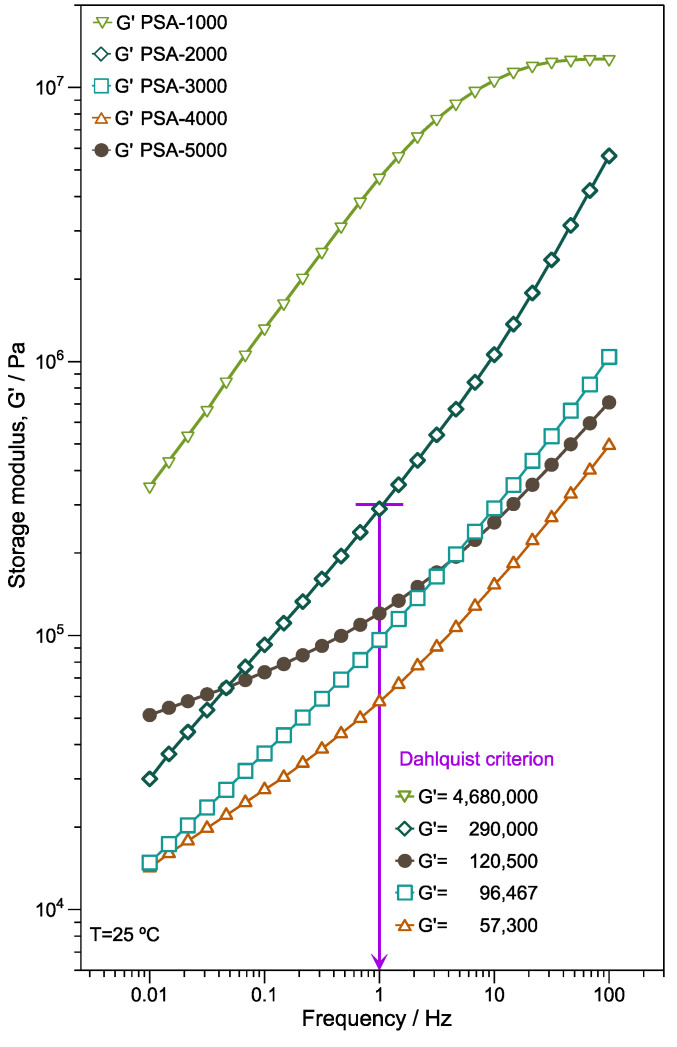
Storage modulus vs. frequency of PSA-1000, PSA-2000, PSA-3000, PSA-4000, and PSA-5000 samples measured at 25 °C.

The rheometer allows the evaluation of the viscoelastic behavior of bulk adhesives, and relate it to its performance in function of its temperature or frequency so that the oscillatory frequency sweep is a valuable tool for characterizing the micro-structure and the bonding and debonding behavior of adhesives [54,55]. Figure 7 illustrates the storage modulus (G’) vs. frequency of the copolymers films. It is important to note that the G’ increases for PSA-1000, PSA-2000, PSA-3000, PSA-4000, and PSA-5000 concerning the frequency exhibiting significant elastic behavior. Except for PSA-5000, there is a trend to decrease G’ around 2 Hz because the *T_g_* reduction influences the polymer chain mobility and softens the films. Compared with PSA-2000, PSA-3000, and PSA-4000, the PSA-5000 sample has high modulus; however, with a tendency to zero at low frequencies (0.1 Hz). In addition, the plateau zone exhibiting at low-medium frequencies indicates a significantly cross-linked structure that is consistent with the gel content of PSA-5000.

There are different principles to correlate the rheological properties with the performance of a PSA. In 1969, Dahlquist introduced one of the key criteria employed currently. The criterion established that to have good “quick tack” performance storage modulus G’ should not exceed 3 × 10^5^ Pa measured at 1 Hz [56]. Figure 7 shows a purple line indicating this measure, also showing the results for each sample. This principle is a starting point that would predict the behavior of adherence. However, it does not consider the rheological frequency domain where the binding and detachment processes occur.

#### 3.5.2. Viscoelastic Windows

The viscoelastic windows (VW) are another principle that illustrates the potential applications of PSA. Unlike the Dahlquist criterion, which only evaluates module G′, VW considers the G′ and the loss module (G″). Chang proposed the construction of viscoelastic windows in 1991 and is made from the values G′ and G” vs. angular frequency. The VW are constructed using four points on the graph: (1) G′ at 0.01 rad/s, G” at 0.01 rad/s; (2) G′ at 0.01 rad/s, G″ at 100 rad/s; (3) G′ at 100 rad/s, G″ at 0.01 rad/s; and (4) G′ at 100 rad/s, G″ at 100 rad/s. In addition, this graph is divided into four quadrants and a central region [57,58]. It can be classified from the location and shape of the VW of the analyzed PSA.

**Figure 8:**
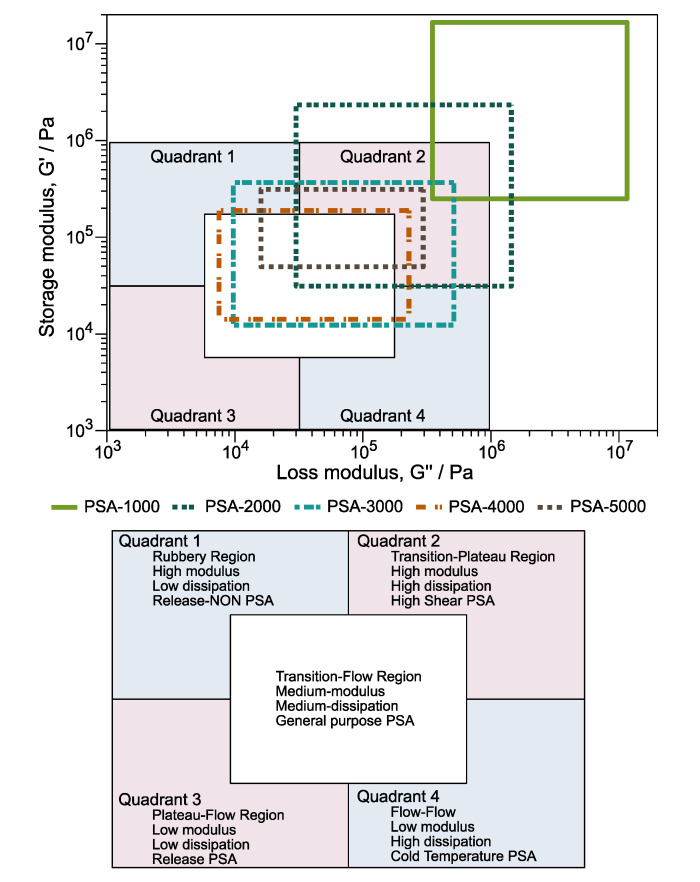
Evaluation of PSA-1000, PSA-2000, PSA-3000, PSA-4000, and PSA-5000 samples under the criteria of viscoelastic windows.

Moreover, the VW chart illustrates the potential applications for PSA considering G′ and G″. Figure 8 shows the VW of PSA-1000, PSA-2000, PSA-3000, PSA-4000, and PSA-5000 samples. It is important to note that the window of PSA-1000 (Poly(DBI_99_-*stat-*IA_1_)) is placed outside of the VW chart because it has the *T_g_* higher than the other copolymers. Meanwhile, the performance of PSA-2000 is localized in quadrant 2, corresponding to high shear, high modulus, and high dissipation. However, according to the Dahlquist criterion is at the limit of the reference value. The evaluation of PSA-3000 is complex because it is positioned in quadrants 1, 2, 4, and the central region. Nonetheless, 60% of the area is located within the central part is classified as general-purpose PSA. Likewise, PSA-5000 presents a window size that interacts with quadrant 1, quadrant 2, and central. The behavior of PSA-4000 is surprising; its values agree in the center of the graph, suggesting that it has a high potential as general-purpose PSA.

### 3.6. Adhesive Properties of Copolymers

The adhesive properties (peel strength, loop tack, and shear strength) of the PSA-2000, PSA-3000, and PSA-4000 were evaluated in order to identify which ratio of DBI:LMA is optimal. Peel strength is the force required to peel away a strip of PSA coated material according to dimensions, speed, and peeling angle of the standard procedure, ASTM D3330. Tack is the ability of the PSA to wet the surface that it contacts. Finally, shear strength is the ability of a material to resist lateral forces. However, these tests are sensitive to several factors, including temperature, adhered surface properties, and are measured utilizing several methods.

**Figure 9:**
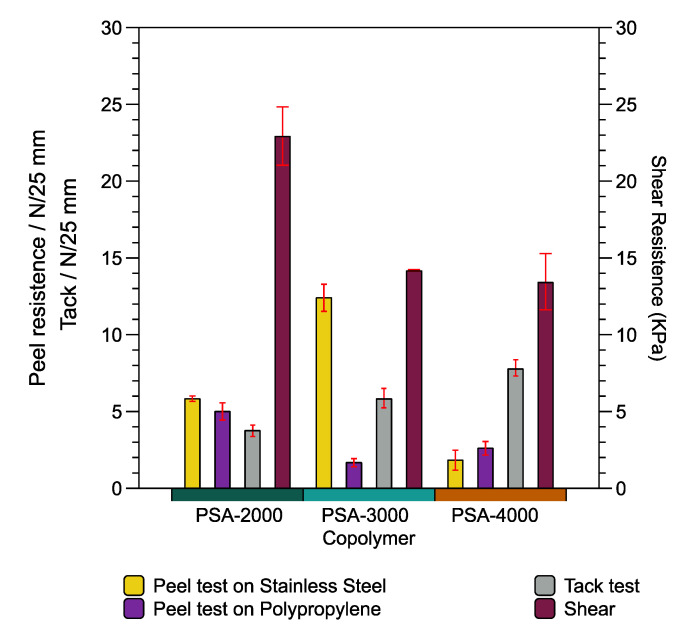
Adhesive properties of PSA-2000, PSA-3000, and PSA-4000 copolymers.

Figure 9 presents the different adhesive properties such as peel test, tack test, and shear for PSA-2000, PSA-3000, and PSA-4000 was performed. The maximum and minimum values of the peel resistance on the stainless steel substrate (yellow color) are observed in the formulation PSA-3000 (DBI:LMA 49:50) and PSA-4000 (DBI:LMA 24:75), respectively. Likewise, the peel resistance on the polypropylene substrate (purple color) decreases concerning the LMA content because the copolymer has a more entangled structure reducing the peel strength.

On the contrary, the tack test (gray color) suggests an increase of values about LMA content. In addition, the behavior of the shear test presents significant magnitude depending on the DBI content. To summarize, the peel and tack results indicate that it is necessary LMA; however, the shear test requires high DBI content. The PSA-2000 and PSA-3000 formulation development present a cohesive failure; in other words, it leaves residues. Meanwhile, PSA 4000 has a type of failure adhesively.

Comparison of the findings with those of other studies (biobased copolymers) confirms similar values. For example, the poly(tetrahydrogeranyl acrylate)-*co*-poly(menthyl methacrylate)-*co*-poly(acrylic acid) copolymer registers magnitudes of 7.7 N/25 mm and 5.1 N/25 mm for peel (stainless steel) and tack test, respectively. In addition, the composition of the copolymer is 84:14:2 wt % [11]. Consider as another example, the poly(2-octyl acrylate)-*co*-poly(isobornyl methacrylate)-*co*-poly(methacrylic acid) copolymer has a value of 6.7 (peel test, N/25 mm), under the following monomer ratio 84:15:1 wbm% (weight % based on total monomer content) [12].

## 4. Conclusions

In the present study, fully biobased waterborne copolymers from itaconic acid (IA), di-*n*-butyl itaconate (DBI), and lauryl methacrylate (LMA) monomers were successfully synthesized via an environmentally friendly process as emulsion polymerization. Various copolymers were obtained with a different DBI:LMA ratio and the identical quantity of IA (1 wt %). The spectroscopic techniques, NMR and FTIR, confirmed that the final composition was close to that of the theoretical formulation, indicating the incorporation of the monomers in the copolymer chain. The DBI:LMA ratio was 99.65:0, 68.17:31.45, 47.82:52.05, 23.53:76.18, and 0:99.32 for PSA-1000 (Poly(DBI_99_-*stat-*IA_1_), PSA-2000 (Poly(DBI_74_-*stat-*LMA_25_-*stat-*IA_1_)), PSA-3000 (Poly(DBI_49_-*stat-*LMA_50_-*stat-*IA_1_)), PSA-4000 (Poly(DBI_24_-*stat-*LMA_75_-*stat-*IA_1_)), and PSA-5000 (Poly(LMA_99_-*stat-*IA_1_)), respectively. The IA value, in the same order, was 0.35, 0.38, 0.13, 0.29, and 0.68 wt %. Copolymer films displayed a single glass transition temperature that suggests a statistical random polymerization. In addition, a branched and cross-linked microstructure was observed in the copolymers (PSA-2000, PSA-3000, PSA-4000, and PSA-5000) owing to the LMA monomer in the emulsion polymerization, provided by the increase in gel content and high molecular weight. The findings of this study of copolymer ratio on rheological and adhesive properties as a first approach also demonstrate the feasibility of utilizing the IA, DBI, and LMA monomers as pressure-sensitive adhesives (PSA). The PSA-4000 presented balanced equilibrium properties and was classified as a general-purpose PSA. Copolymer films with more than 50% DBI in the formulation may be useful in adhesive applications outside PSA. However, there is a possibility of improving the formulation because no cross-linking or additives were used.

## Figures and Tables

**Table 1:** Theoretical quantities of the latex dispersions.

Sample ID	Copolymer	Composition ^1^ DBI: LMA: IA			DBI (g)	LMA (g)	IA (g)
PSA-1000	Poly(DBI_99_-*stat-*IA_1_)	99	0	1	22.8	0	0.23
PSA-2000	Poly(DBI_74_-*stat-*LMA_25_-*stat-*IA_1_)	74	25	1	16.9	5.7	0.23
PSA-3000	Poly(DBI_49_-*stat-*LMA_50_-*stat-*IA_1_)	49	50	1	11.2	11.4	0.23
PSA-4000	Poly(DBI_24_-*stat-*LMA_75_-*stat-*IA_1_)	24	75	1	5.5	17.1	0.23
PSA-5000	Poly(LMA_99_-*stat-*IA_1_)	0	99	1	0	22.6	0.23

^1^ All formulations contain DI-H20 66.25 g; SDS 0.95 g; KPS 0.23 g, and NH_3_OH 2.25 mL (1 M).

**Table 2:** Comparison of theoretical vs. NMR ratios.

Sample ID	Copolymer	Theoretical (wt %) DBI:LMA:IA			NMR (wt %) DBI:LMA:IA		
PSA-1000	Poly(DBI_99_-*stat-*IA_1_)	99	0	1	99.65	0	0.35
PSA-2000	Poly(DBI_74_-*stat-*LMA_25_-*stat-*IA_1_)	74	25	1	68.17	31.45	0.38
PSA-3000	Poly(DBI_49_-*stat-*LMA_50_-*stat-*IA_1_)	49	50	1	47.82	52.05	0.13
PSA-4000	Poly(DBI_24_-*stat-*LMA_75_-*stat-*IA_1_)	24	75	1	23.53	76.18	0.29
PSA-5000	Poly(LMA_99_-*stat-*IA_1_)	0	99	1	0	99.32	0.68

**Table 3:** Comparison of theoretical versus experimental glass transition temperature obtained by DSC.

Sample ID	Copolymer	Theoretical ^1^ °C	Experimental °C
PSA-1000	Poly(DBI_99_-*stat-*IA_1_)	14.2	19.3
PSA-2000	Poly(DBI_74_-*stat-*LMA_25_-*stat-*IA_1_)	−16.4	−10.5
PSA-3000	Poly(DBI_49_-*stat-*LMA_50_-*stat-*IA_1_)	−33.3	−23.2
PSA-4000	Poly(DBI_24_-*stat-*LMA_75_-*stat-*IA_1_)	−50.3	−53.4
PSA-5000	Poly(LMA_99_-*stat-*IA_1_)	−64.4	n.d.

^1^ Calculated with Fox equation.

## Data Availability

The data presented in this study are available in the Supplementary Materials.

## Supplementary Materials

The following supporting information can be downloaded at: https://www.mdpi.com/article/10.3390/polym14030632/s1, Figure S1: Mechanisms of particle nucleation formation; Figure S2: ^1^H-NMR spectrum of poly(DBI-*stat*-AI) (99 wt % DBI: 1 wt % AI) in CDCl_3_; Figure S3: ^1^H-NMR spectrum of PSA-5000 (poly(LMA-*stat*-IA) (99 wt % LMA: 1 wt % IA)) in CDCl3; Figure S4: ^1^H-NMR spectrum of PSA 3000 (poly(DBI-stat-LMA-*stat*-IA) (49 wt % DBI: 50 wt % LMA: 1 wt % IA)) in CDCl_3_; Figure S5: ^1^H-^1^H COSY of PSA-3000 (poly(DBI-*stat*-LMA-*stat*-IA) (49 wt % DBI: 50 wt % LMA: 1 wt % IA)) in CDCl_3_; Figure S6: ^1^H-^1^H COSY of PSA-2000 (poly(DBI-*stat*-LMA-*stat*-IA) (74 wt % DBI: 25 wt % LMA: 1 wt % IA)) in CDCl_3_; Figure S7: ^1^H-^1^H COSY of PSA-4000 (poly(DBI-*stat*-LMA-*stat*-IA) (24 wt % DBI: 75 wt % LMA: 1 wt % IA)) in CDCl_3_; Figure S8: ^1^H-^13^C HSQC of PSA-3000 (poly(DBI-*stat*-LMA-*stat*-IA) (49 wt % DBI: 50 wt % LMA: 1 wt % IA)) in CDCl_3_; Figure S9: ^1^H-^13^C HSQC of PSA-2000 (poly(DBI-*stat*-LMA-*stat*-IA) (74 wt % DBI: 25 wt % LMA: 1 wt % IA)) in CDCl_3_; Figure S10: ^1^H-^13^C HSQC of PSA-4000 (poly(DBI-*stat*-LMA-*stat*-IA) (24 wt % DBI: 75 wt % LMA: 1 wt % IA)) in CDCl_3_; Figure S11: ^13^C-NMR spectrum of PSA 3000 (poly(DBI-*stat*-LMA-*stat*-IA) (49 wt % DBI: 50 wt % LMA: 1 wt % IA)) in CDCl_3_; Figure S12: DEPT-135 of PSA-3000 sample; Figure S13: Analysis of PSA-3000 at different relaxation times (D1); Table S1: Area and number of protons used to calculate the composition of copolymers; Figure S14: Area correlation of the band 2923 cm^−1^ as a function of the LMA content; Figure S15: Area correlation of the band 2853 cm^−1^ as a function of the LMA content; Figure S16: FTIR spectra of copolymers films. (a) PSA-5000; (b) PSA-4000; (c) PSA-3000; (d) PSA-2000; (e) PSA-1000; Figure S17: Kinetic conversion study in relation to the gel content of the PSA-4000 sample; Figure S18: DSC thermograms of copolymers films. (a) PSA-5000; (b) PSA-4000; (c) PSA-3000; (d) PSA-2000; (e) PSA-1000.
