# Supplementary material for: Dibutyl Itaconate and Lauryl Methacrylate Copolymers by Emulsion Polymerization for Development of Sustainable Pressure-Sensitive Adhesives
Source: Polymers (Basel). 2022 Feb 7;14(3):632. doi: 10.3390/polym14030632 (PMC8840584; doi:10.3390/polym14030632)
Supplement: Supplementary file 1 [file polymers-14-00632-s001.zip › polymers-1482761-supplementary.pdf]

polymers

MDPI

Supplementary Materials

Dibutyl Itaconate and Lauryl Methacrylate Copolymers by Emulsion Polymerization for Development of Sustainable Pressure-Sensitive Adhesives

Carlos Rafael Casas-Soto [1], Alain Salvador Conejo-Dávila [1], Velia Osuna [2], David Chávez-Flores [3], José Carlos Espinoza-Hicks [3], Sergio Gabriel Flores-Gallardo [1] and Alejandro Vega-Rios [1,*]

[1] Departament of Engineering and Materials Chemistry, Centro de Investigación en Materiales Avanzados, SC, Miguel de Cervantes No. 120, Chihuahua C.P. 31136, Mexico; carlos.casas@cimav.edu.mx (C.R.C.-S.); alain.conejo@cimav.edu.mx (A.S.C.-D.); sergio.flores@cimav.edu.mx (S.G.F.-G.)

[2] Consejo Nacional de Ciencia y Tecnología (CONACyT)-Centro de Investigación en Materiales Avanzados, SC (CIMAV), Miguel de Cervantes No. 120, Chihuahua C.P. 31136, Mexico; velia.osuna@cimav.edu.mx

[3] Facultad de Ciencias en Química, Universidad Autonóma de Chihuahua, Chihuahua C.P. 31125, Mexico; dchavezf@uach.mx (D.C.-F.); jhicks@uach.mx (J.C.E.-H.)

* Correspondence: alejandro.vega@cimav.edu.mx; Tel.: +52-01-614-439-4831

Citation: Casas-Soto, C.R.; Conejo-Dávila, A.S.; Osuna, V.; Chávez-Flores, D.; Espinoza-Hicks, J.C.; Flores-Gallardo, S.G.; Vega-Rios, A. Dibutyl Itaconate and Lauryl Methacrylate Copolymers by Emulsion Polymerization for Development of Sustainable Pressure-Sensitive Adhesives. *Polymers* **2022**, *14*, 632. https://doi.org/10.3390/xxxxx

Academic Editors: Charles Frihart, Christopher G. Hunt and Ahmed I. A. Abd El-Mageed

Received: 11 November 2021
Accepted: 3 February 2022
Published: date

Publisher's Note: MDPI stays neutral with regard to jurisdictional claims in published maps and institutional affiliations.

Copyright: © 2022 by the authors. Licensee MDPI, Basel, Switzerland. This article is an open access article distributed under the terms and conditions of the Creative Commons Attribution (CC BY) license (http://creativecommons.org/licenses/by/4.0/).

List of Figures

Figure S1. Mechanisms of particle nucleation formation. 2
Figure S2. ^{1}H-NMR spectrum of PSA-1000 [poly(DBI-stat-IA) (99 wt. % DBI : 1 wt.% IA)] in CDCl3. 3
Figure S3. ^{1}H-NMR spectrum of PSA-5000 [poly(LMA-stat-IA) (99 wt. % LMA: 1 wt.% IA)] in CDCl3. 3
Figure S4. ^{1}H-NMR spectrum of PSA 3000 [poly(DBI-stat-LMA-stat-IA) (49 wt. % DBI : 50 wt. % LMA: 1 wt.% IA)] in CDCl3. 4
Figure S5. ^{1}H-^{1}H COSY of PSA-3000 [poly(DBI-stat-LMA-stat-IA) (49 wt. % DBI : 50 wt. % LMA: 1 wt.% IA)] in CDCl3. 4
Figure S6. ^{1}H-^{1}H COSY of PSA-2000 [poly(DBI-stat-LMA-stat-IA) (74 wt. % DBI : 25 wt. % LMA: 1 wt.% IA)] in CDCl3. 5
Figure S7. ^{1}H-^{1}H COSY of PSA-4000 [poly(DBI-*stat*-LMA-*stat*-IA) (24 wt. % DBI : 75 wt. % LMA: 1 wt.% IA)] in CDCl3. 5
Figure S8. ^{1}H-^{13}C HSQC of PSA-3000 [poly(DBI-stat-LMA-stat-IA) (49 wt. % DBI : 50 wt. % LMA: 1 wt.% IA)] in CDCl3. 6
Figure S9. ^{1}H-^{13}C HSQC of PSA-2000 [poly(DBI-stat-LMA-stat-IA) (74 wt. % DBI : 25 wt. % LMA: 1 wt.% IA)] in CDCl3. 6
Figure S10. ^{1}H-^{13}C HSQC of PSA-4000 [poly(DBI-*stat*-LMA-*stat*-IA) (24 wt. % DBI : 75 wt. % LMA: 1 wt.% IA)] in CDCl3. 7
Figure S11. ^{13}C-NMR spectrum of PSA 3000 [poly(DBI-stat-LMA-stat-IA) (49 wt. % DBI : 50 wt. % LMA: 1 wt.% IA)] in CDCl3. 7
Figure S12. DEPT-135 of PSA-3000 sample. 8
Figure S13. Analysis of PSA-3000 at different relaxation times (D1)... 8

Figure S14. Area correlation of the band 2923 cm^{-1} as a function of the LMA content. 10
Figure S15. Area correlation of the band 2853 cm^{-1} as a function of the LMA content. 10
Figure S16. FTIR spectra of copolymers films. a) PSA-5000; b) PSA-4000; c) PSA-3000; d) PSA-2000; e) PSA-1000. 11
Figure S17. Kinetic conversion study in relation to the gel content of the PSA-4000 sample. 11
Figure S18. DSC thermograms of copolymers films. a) PSA-5000; b) PSA-4000; c) PSA-3000; d) PSA-2000; e) PSA-1000. 12

List of Tables

Table S1. Area and number of protons used to calculate the composition of copolymers. 9

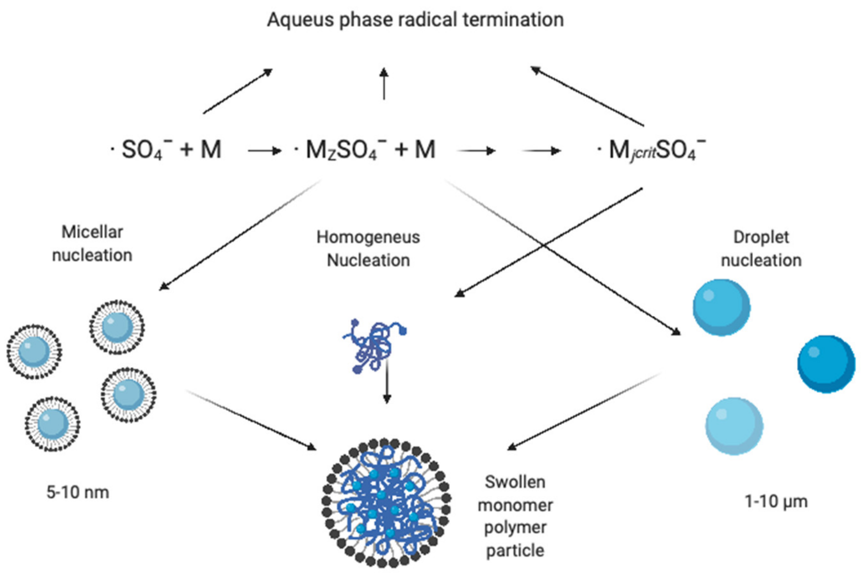

Figure S1. Mechanisms of particle nucleation formation.

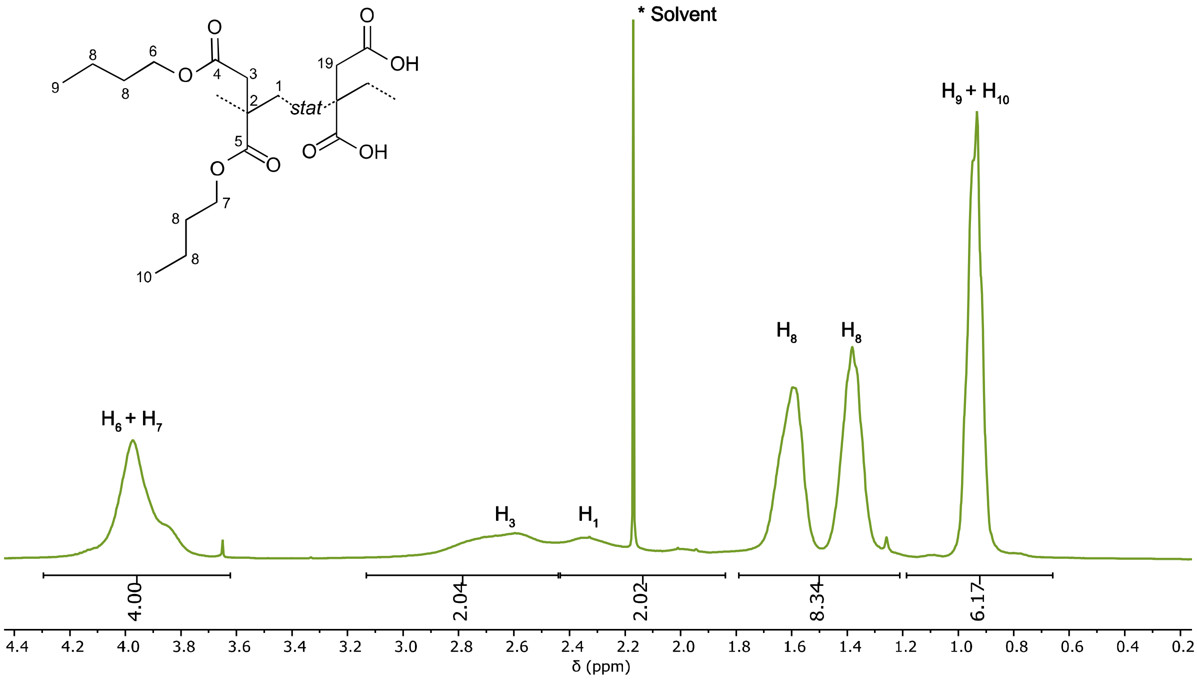

Figure S2. ^{1}H-NMR spectrum of PSA-1000 [poly(DBI-stat-IA) (99 wt. % DBI : 1 wt.% IA)] in $CDCl_3$.

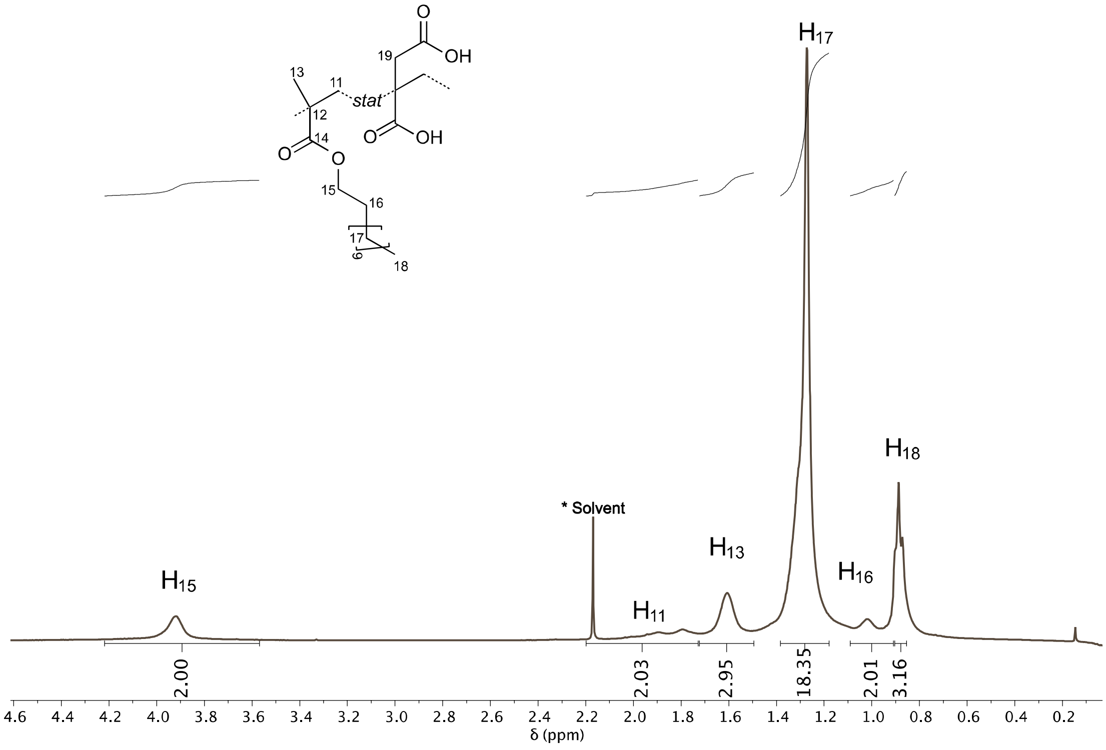

Figure S3. ^{1}H-NMR spectrum of PSA-5000 [poly(LMA-stat-IA) (99 wt. % LMA: 1 wt.% IA)] in CDCl3.

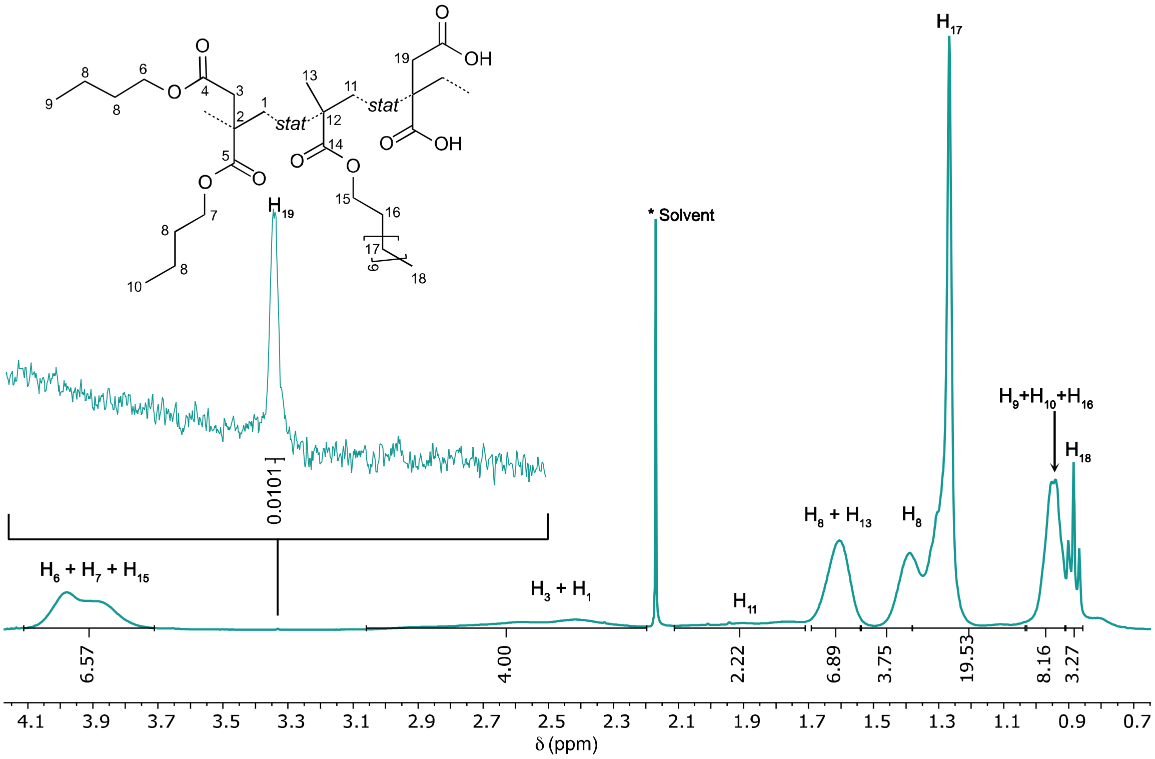

Figure S4. ^{1}H-NMR spectrum of PSA 3000 [poly(DBI-stat-LMA-stat-IA) (49 wt. % DBI : 50 wt. % LMA: 1 wt.% IA)] in CDCl3.

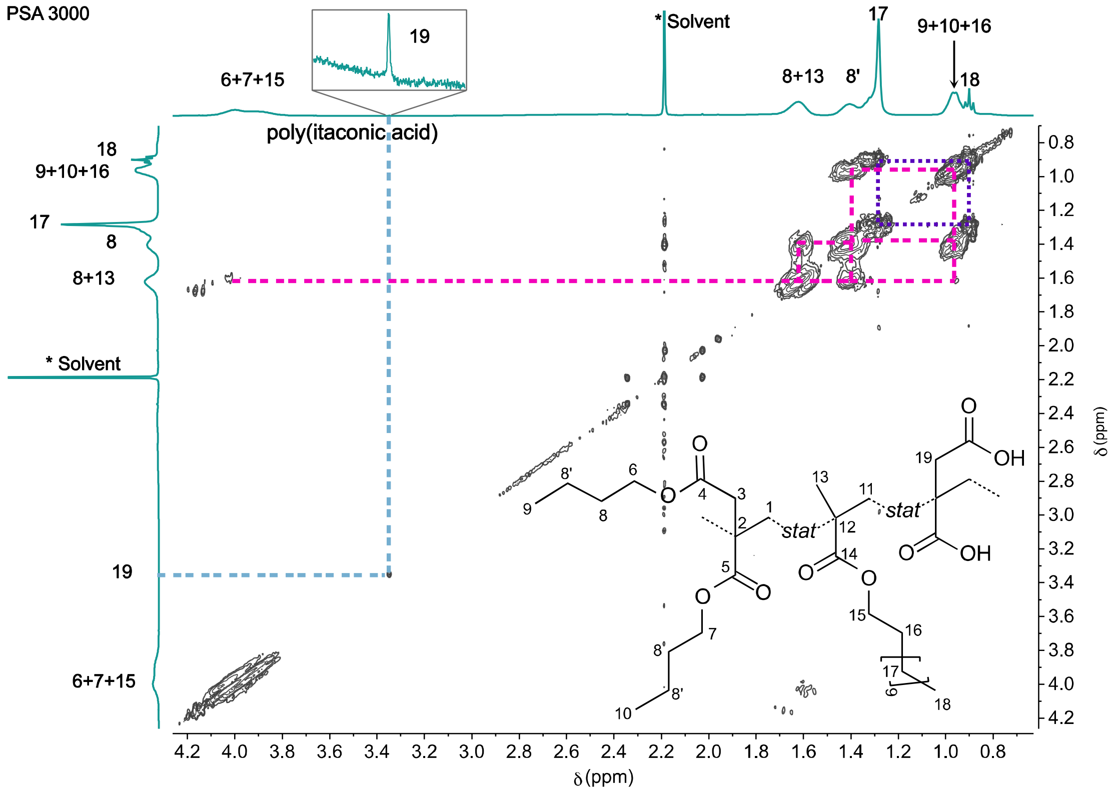

Figure S5. ^{1}H-^{1}H COSY of PSA-3000 [poly(DBI-stat-LMA-stat-IA) (49 wt. % DBI : 50 wt. % LMA: 1 wt.% IA)] in CDCl3.

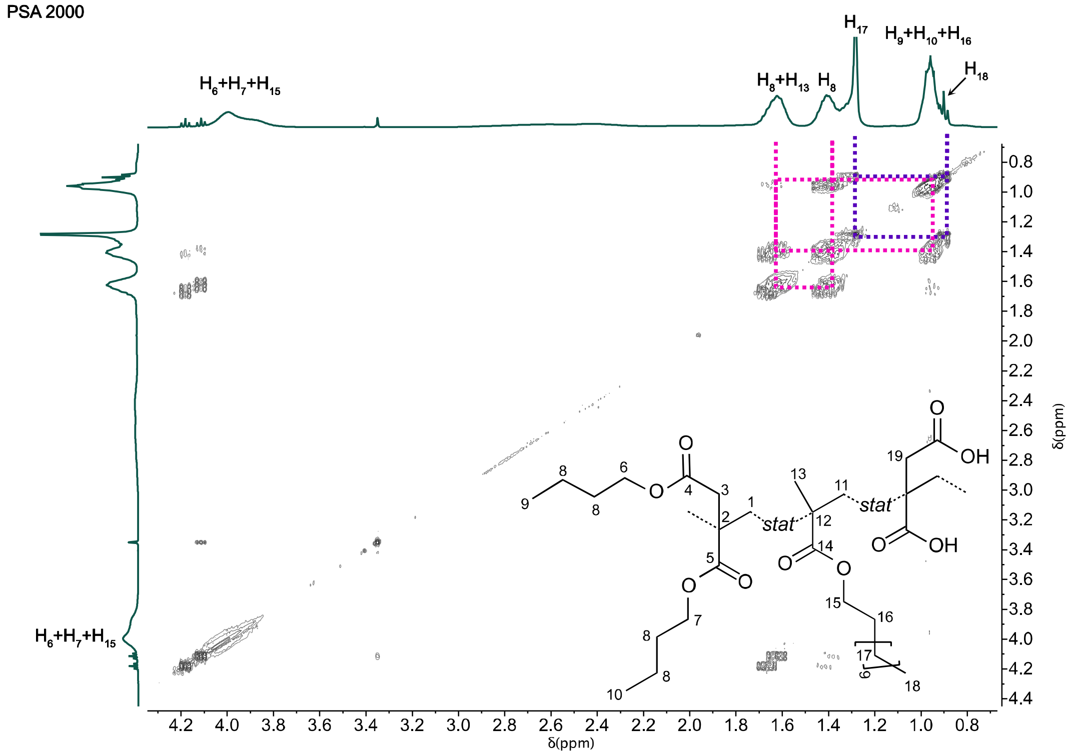

Figure S6. ^{1}H-^{1}H COSY of PSA-2000 [poly(DBI-stat-LMA-stat-IA) (74 wt. % DBI : 25 wt. % LMA: 1 wt.% IA)] in $CDCl_3$.

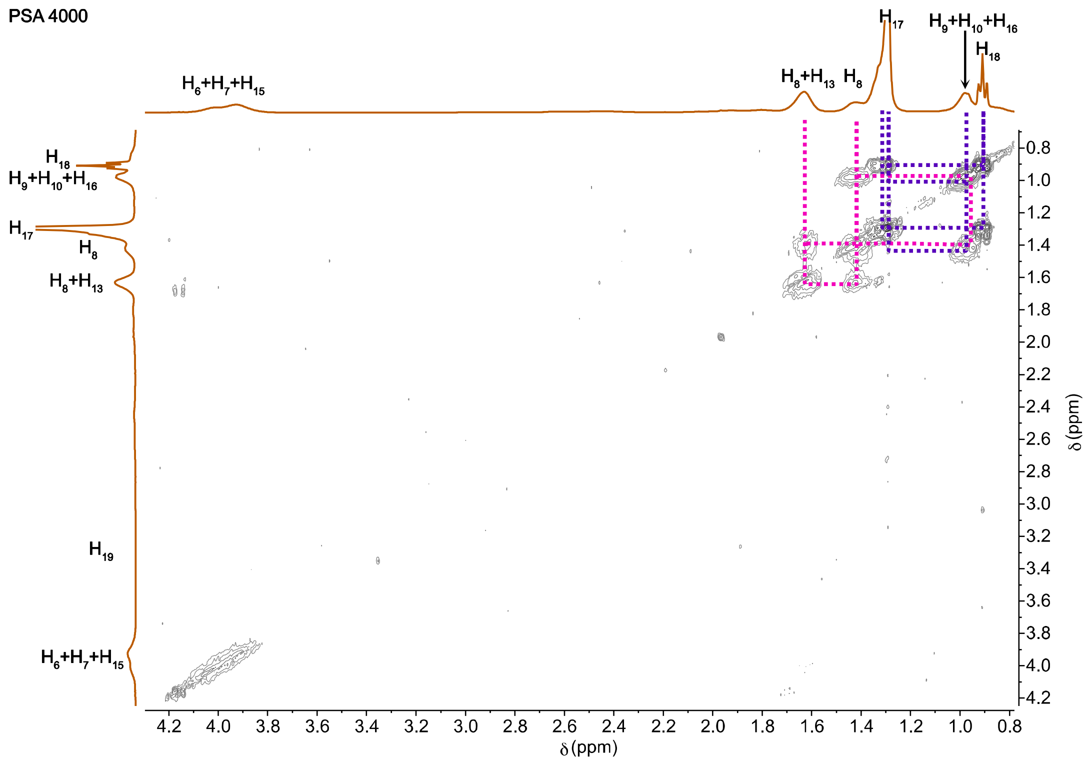

Figure S7. ^{1}H-^{1}H COSY of PSA-4000 [poly(DBI-*stat*-LMA-*stat*-IA) (24 wt. % DBI : 75 wt. % LMA: 1 wt.% IA)] in $CDCl_3$.

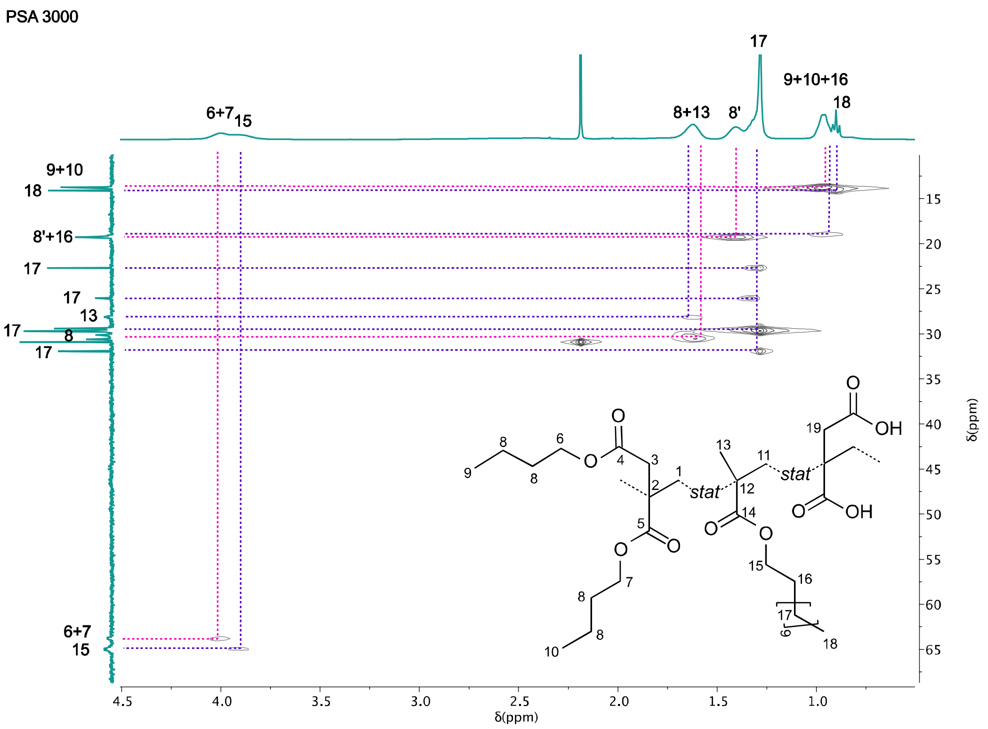

Figure S8. ^1H-^{13}C HSQC of PSA-3000 [poly(DBI-stat-LMA-stat-IA) (49 wt. % DBI : 50 wt. % LMA: 1 wt.% IA)] in $CDCl_3$.

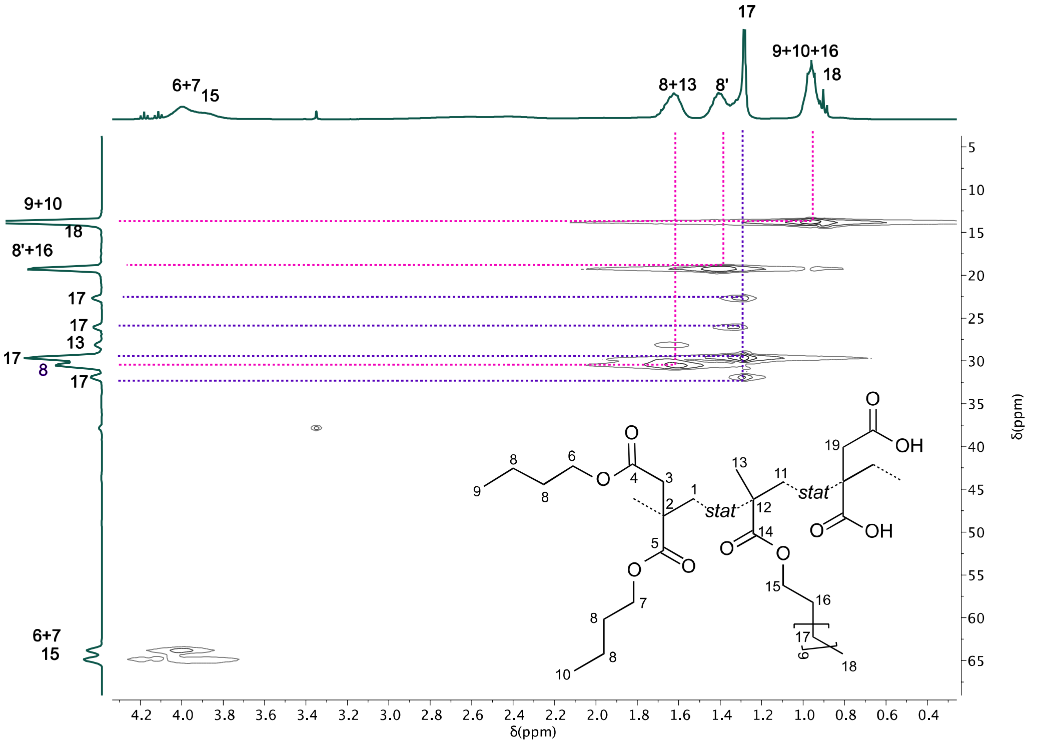

Figure S9. ^1H-^{13}C HSQC of PSA-2000 [poly(DBI-stat-LMA-stat-IA) (74 wt. % DBI : 25 wt. % LMA: 1 wt.% IA)] in $CDCl_3$.

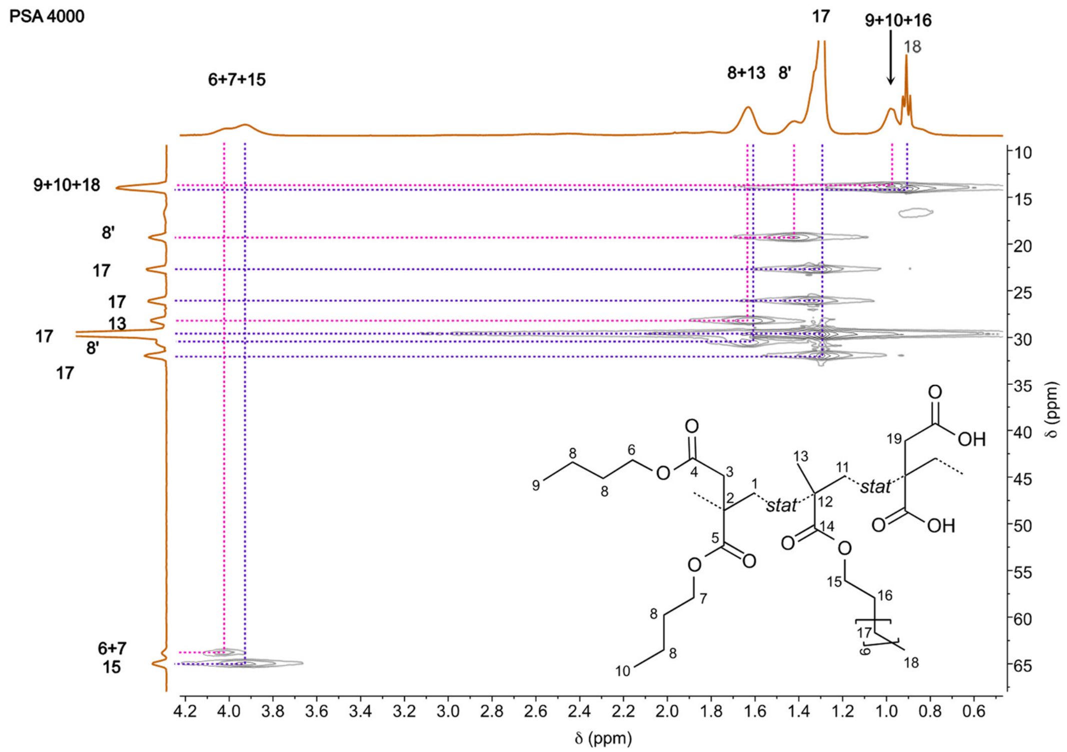

Figure S10. ^1H-^{13}C HSQC of PSA-4000 [poly(DBI-*stat*-LMA-*stat*-IA) (24 wt. % DBI : 75 wt. % LMA: 1 wt.% IA)] in $CDCl_3$.

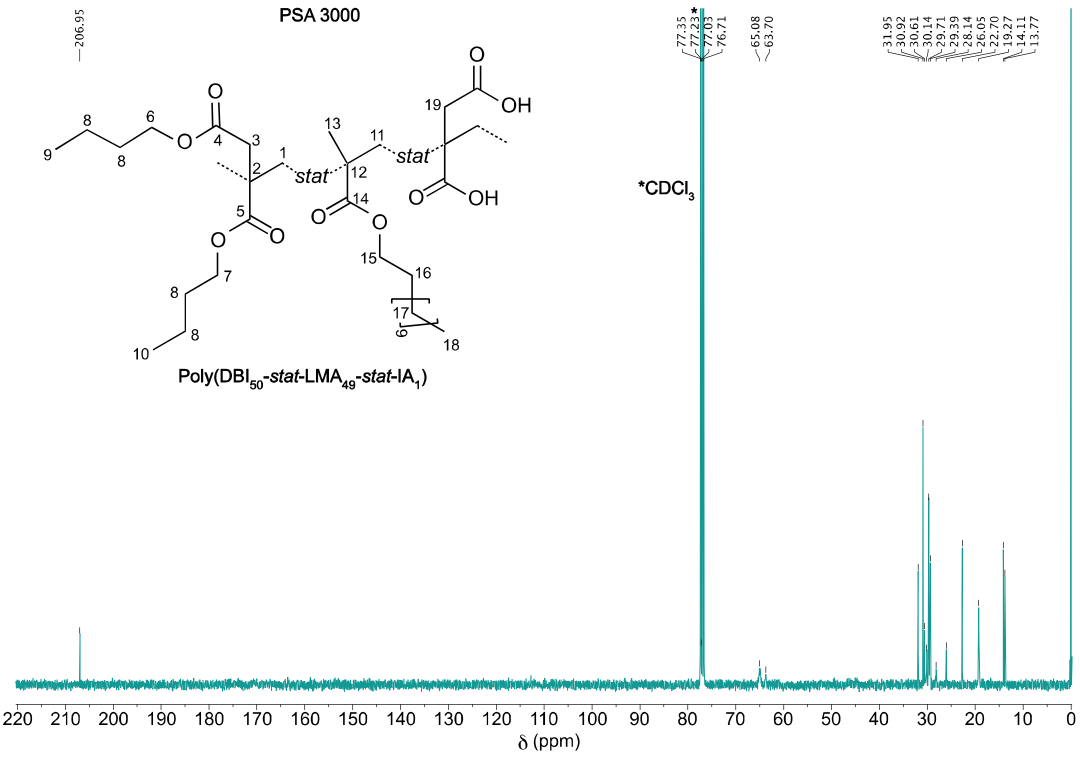

Figure S11. ^{13}C-NMR spectrum of PSA 3000 [poly(DBI-stat-LMA-stat-IA) (49 wt. % DBI : 50 wt. % LMA: 1 wt.% IA)] in $CDCl_3$.

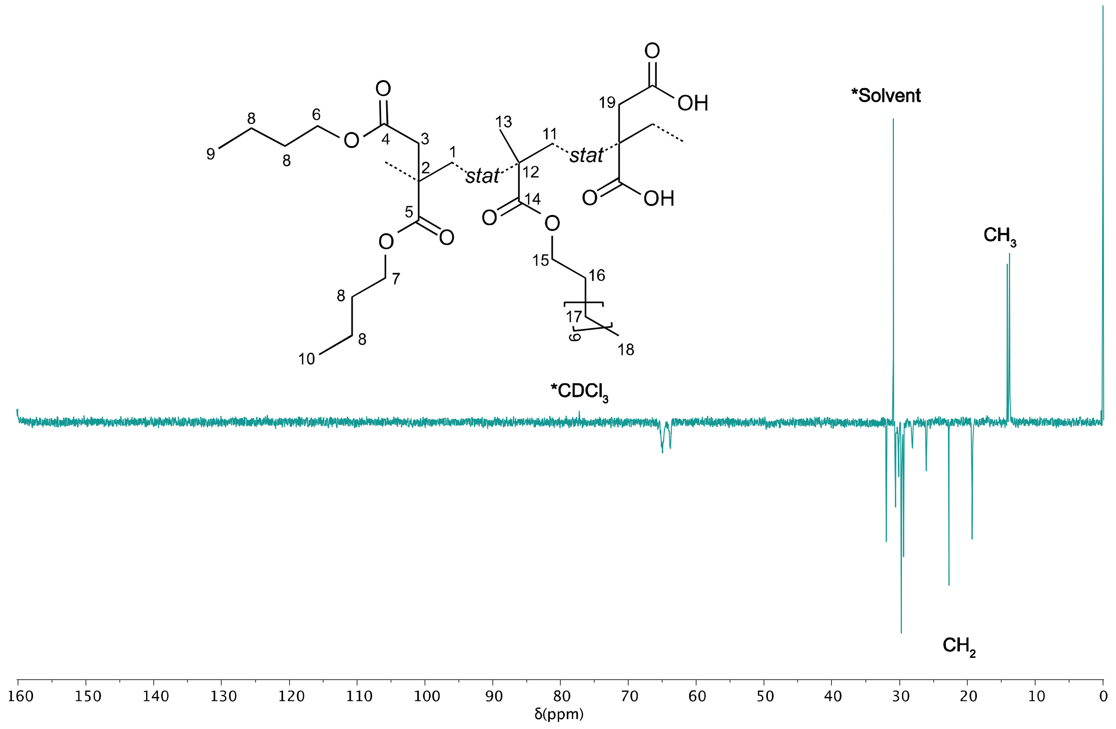

Figure S12. DEPT-135 of PSA-3000 sample.

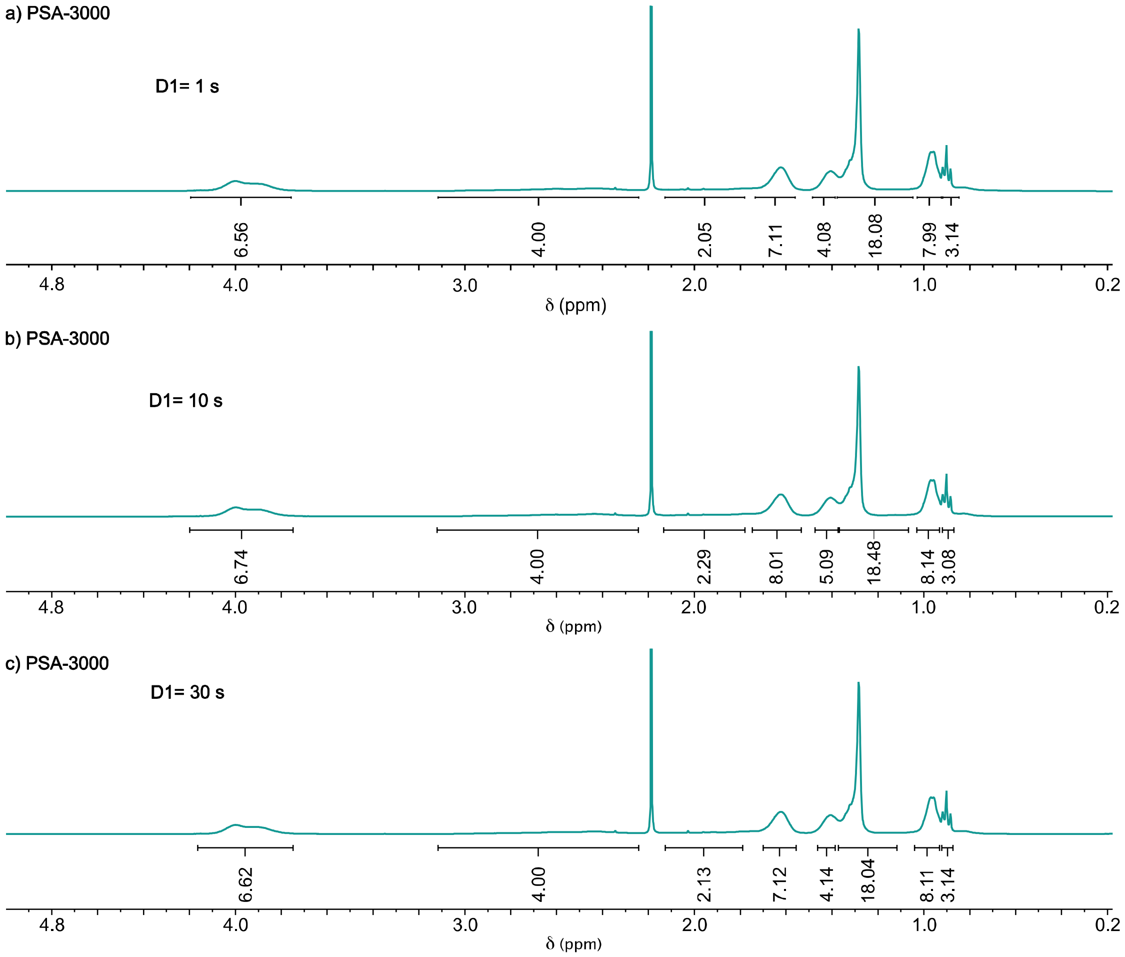

Figure S13. Analysis of PSA-3000 at different relaxation times (D1).

Table S1. Area and number of protons used to calculate the composition of copolymers.

Sample	IA		DBI		LMA	
	A_{19}	P 19	A (1+3)	P (1+3)	A_{18}	P 18
**DB1**	0.0070	2	4.0540	4	0	0
**LMMA**	0.0145	2	0	0	3.1556	3
**PDBI 49**	0.0050	2	4.0010	4	3.2662	3
**PDBI 74**	0.0076	2	2.7045	4	0.9360	3
**PDBI 24**	0.0064	2	1.0850	4	2.6337	3

Binary copolymers

$$\% IA = \frac{\frac{A_{19}}{P19}}{\frac{A_{19}}{P19} + \frac{A_{1+3}}{P(1+3)}} \times 100$$

$$\% DBI = \frac{\frac{A_{1+3}}{P(1+3)}}{\frac{A_{19}}{P19} + \frac{A_{1+3}}{P(1+3)}} \times 100$$

$$\% LMMA = \frac{\frac{A_{18}}{P(18)}}{\frac{A_{19}}{P19} + \frac{A_{18}}{P(18)}} \times 100$$

Ternary copolymers

$$\% IA = \frac{\frac{A_{19}}{P19}}{\frac{A_{19}}{P19} + \frac{A_{1+3}}{P(1+3)} + \frac{A_{18}}{P(18)}} \times 100$$

$$\% DBI = \frac{\frac{A_{1+3}}{P(1+3)}}{\frac{A_{19}}{P19} + \frac{A_{1+3}}{P(1+3)} + \frac{A_{18}}{P18}} \times 100$$

$$\% LMMA = \frac{\frac{A_{18}}{P(18)}}{\frac{A_{19}}{P19} + \frac{A_{18}}{P(18)} + \frac{A_{1+3}}{P(1+3)}} \times 100$$

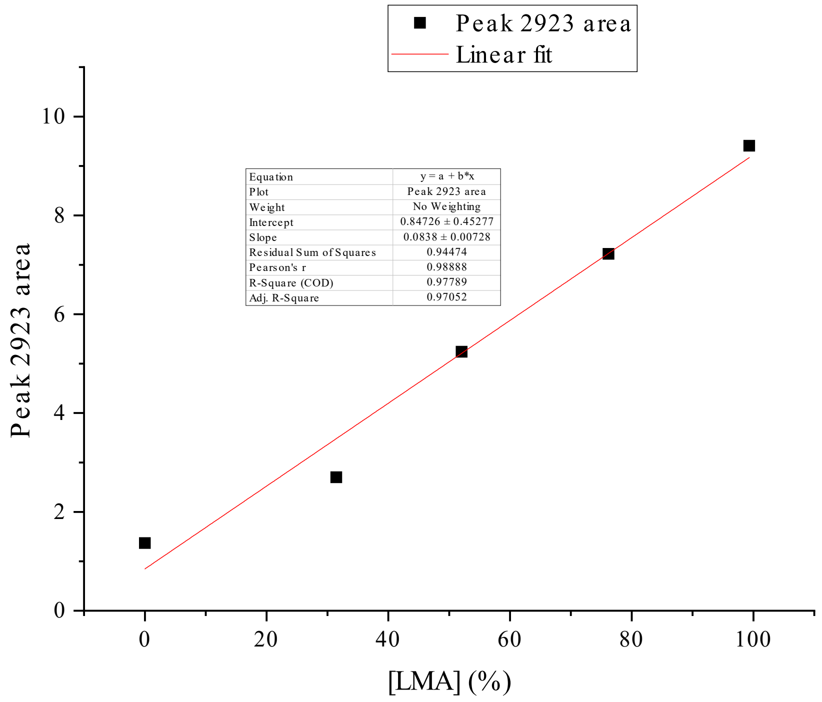

Figure S14. Area correlation of the band 2923 cm^{-1} as a function of the LMA content.

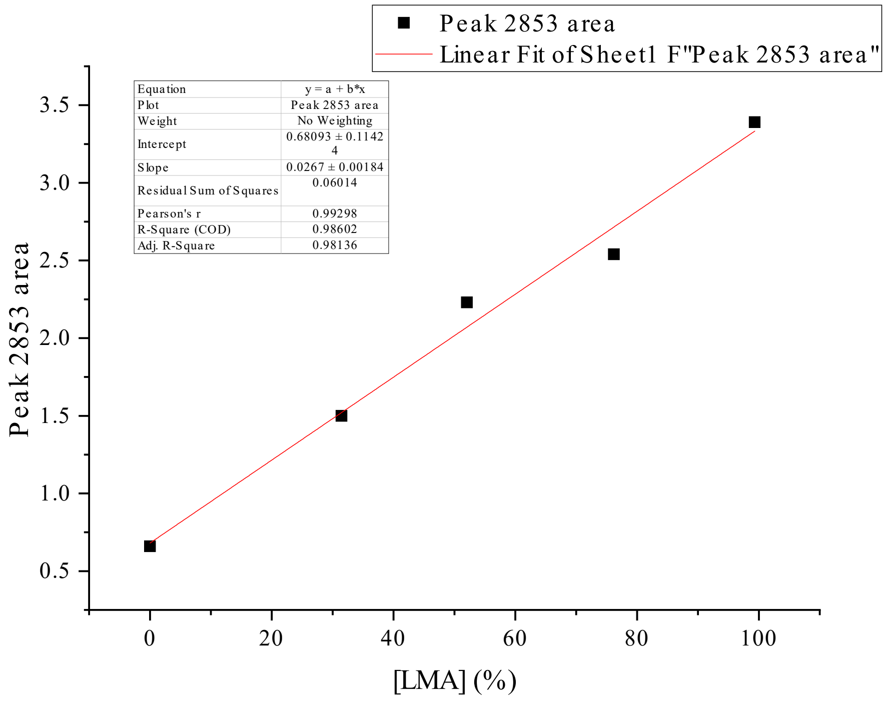

Figure S15. Area correlation of the band 2853 cm^{-1} as a function of the LMA content.

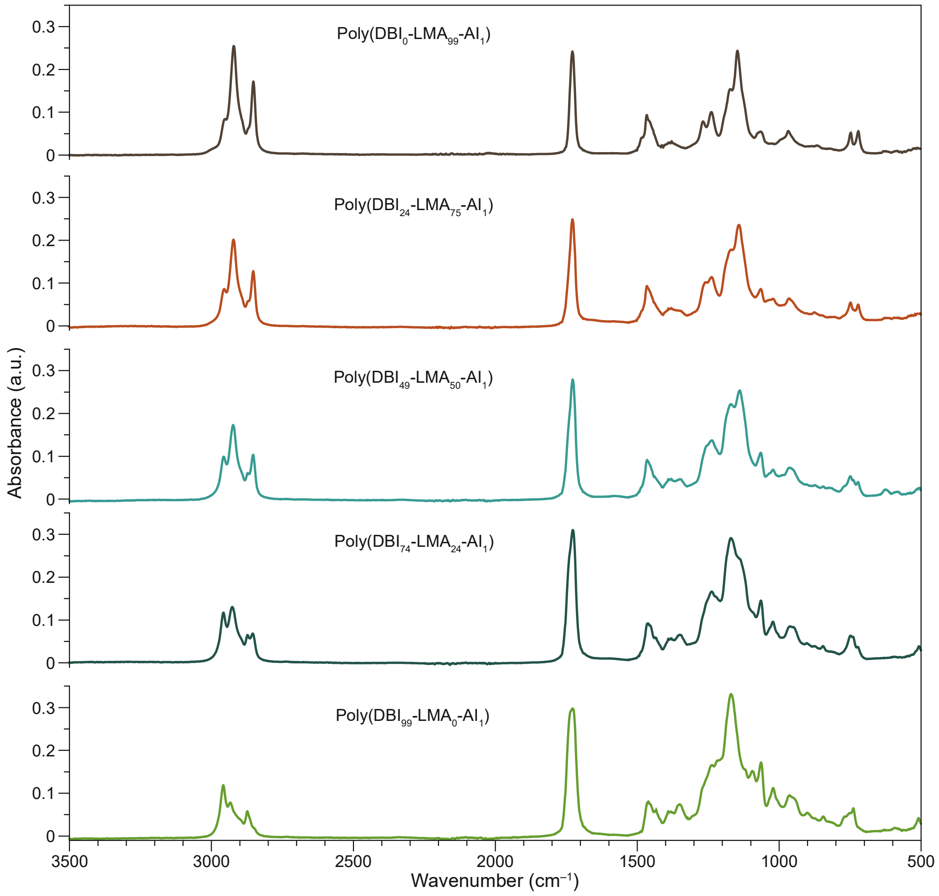

Figure S16. FTIR spectra of copolymers films. a) PSA-5000; b) PSA-4000; c) PSA-3000; d) PSA-2000; e) PSA-1000.

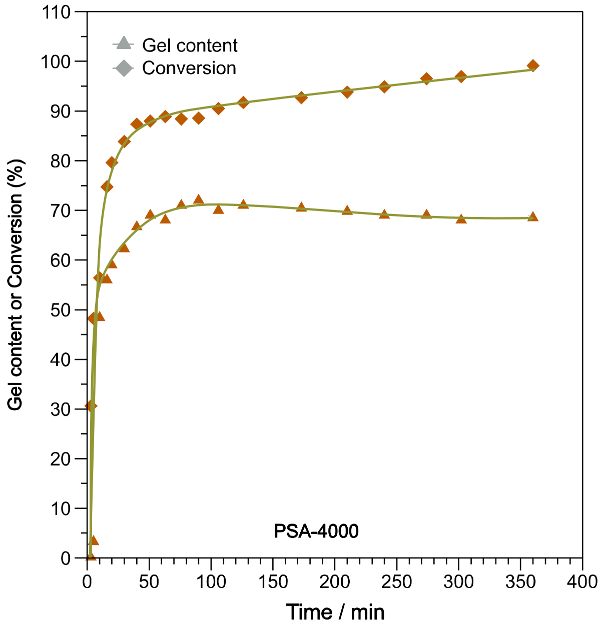

Figure S17. Kinetic conversion study in relation to the gel content of the PSA-4000 sample.

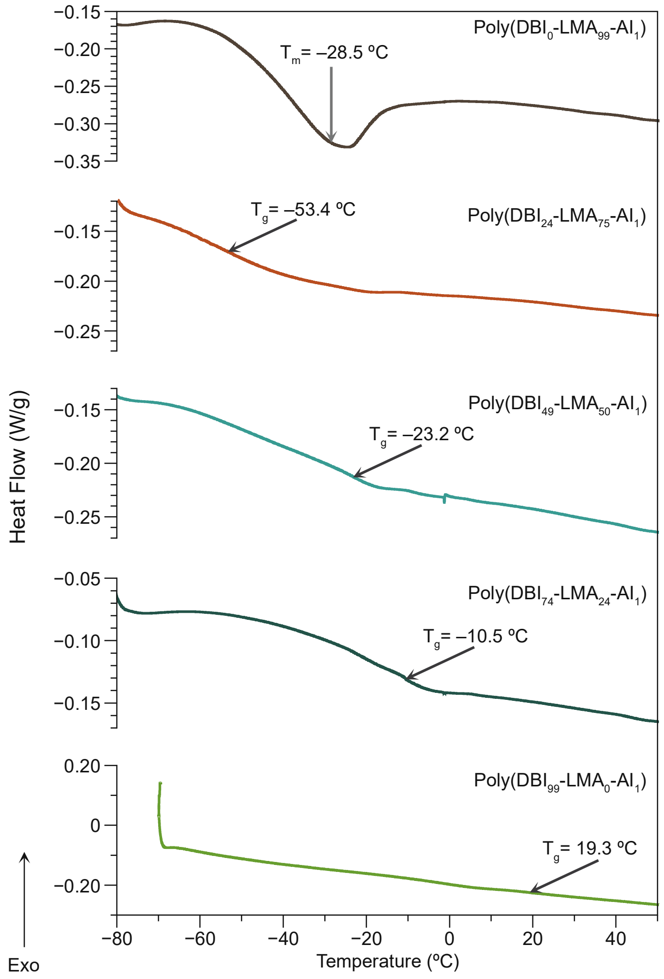

Figure S18. DSC thermograms of copolymers films. a) PSA-5000; b) PSA-4000; c) PSA-3000; d) PSA-2000; e) PSA-1000.
